# Hydrogel microspheres for precision biomedicine: network engineering, microfabrication, and therapeutic applications

**DOI:** 10.1039/d6ra03927h

**Published:** 2026-07-20

**Authors:** Yichen Sun, Zixuan Xiao, Xiaoyuan Zhang, Zhiqiang Su, Xu Wang, Qibiao Wu

**Affiliations:** a Faculty of Chinese Medicine, Macau University of Science and Technology Avenida Wai Long, Taipa Macau 999078 China qbwu@must.edu.mo; b State Key Laboratory of Chemical Resource Engineering, Beijing Key Laboratory of Advanced Functional Polymer Composites, Beijing University of Chemical Technology Beijing 100029 China suzq@mail.buct.edu.cn; c Nanjing University of Chinese Medicine First Clinical Medical College Nanjing 210029 China njzywangxu@126.com; d The Macau Holy House of Mercy Macau 999078 China; e State Key Laboratory of Quality Research in Chinese Medicine, Macau University of Science and Technology Avenida Wai Long, Taipa Macau 999078 China; f Chinese Medicine Guangdong Laboratory (Hengqin Laboratory), Guangdong-Macao ln-Depth Cooperation Zone in Hengqin 519000 P. R. China

## Abstract

Hydrogels and hydrogel microspheres have emerged as highly adaptable biomaterial platforms for precision biomedicine, owing to their hydrated polymer networks, tissue-like physicochemical properties, and capacity to host drugs, biomacromolecules and living cells. In particular, hydrogel microspheres extend the utility of bulk hydrogels by introducing injectability, large interfacial area, modular assembly, tunable microporosity and spatially programmable microenvironments. These features make them attractive for localized drug delivery, cell transplantation, tissue regeneration and emerging therapeutic systems. In this review, we summarize the major physical and chemical strategies used to construct hydrogel networks, and compare representative fabrication methods for hydrogel microspheres, including emulsion polymerization, electrospraying, microfluidics and photolithography. We further discuss how network chemistry, particle geometry, fabrication precision and microsphere assembly influence cargo loading, release behavior, cell compatibility, mechanical performance and *in vivo* functionality. Representative biomedical applications are then reviewed, with emphasis on sustained drug delivery, cell delivery and tissue engineering, as well as emerging uses in soft tissue reconstruction, neural guidance and compartmentalized bioactive systems. Finally, we highlight key challenges that must be addressed for clinical translation, including scalable manufacturing, batch-to-batch reproducibility, sterilization, biosafety, degradation control and application-specific validation. Overall, this review bridges existing research gaps by elucidating the interplay between network chemistry and particle geometry. Furthermore, it establishes criteria for selecting optimal microfabrication techniques, guiding the evolution of hydrogel microspheres from simple delivery carriers to engineered therapeutic microenvironments through the integrated design of materials and bioengineering.

## Abstract 2

This review summarizes the network engineering, microfabrication strategies, biomedical applications, and translational challenges of hydrogel microspheres for precision biomedicine.

## Graphical abstract

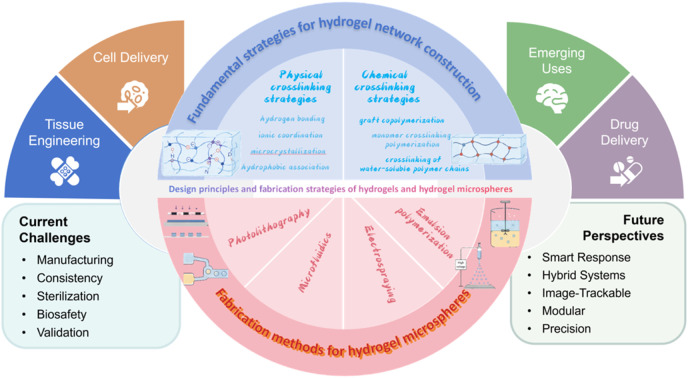

## 1. Introduction

### 1.1 Biomedical relevance of hydrogels

Hydrogels are a major class of soft biomaterials characterized by interconnected three-dimensional polymer networks and high-water content. The topology and organization of these polymer networks strongly influence hydrogel mechanics, permeability, and biological interactions, thereby affecting their biomedical performance.^1^ This combination gives them excellent hydrophilicity, structural adaptability and biological compatibility, making them attractive for tissue engineering, wound repair and drug delivery applications.^2–6^ Particularly in drug delivery systems, hydrogel microspheres can provide localized and sustained release of therapeutic agents while protecting bioactive cargos from rapid degradation, making them promising platforms for precision medicine applications.^7^ Since the pioneering development of hydrogel-based contact lenses in 1960, hydrogels have evolved from simple hydrated polymer matrices into multifunctional biomedical platforms for controlled drug release, implantable materials and scaffold engineering.^8–11^ The soft and water-rich microenvironment of hydrogels can protect encapsulated therapeutic agents and living cells, accommodate biomacromolecules, and support cellular functions, thereby providing a versatile platform for localized therapy and regenerative medicine.

The biomedical value of hydrogels is particularly evident in diseases that require spatially controlled and sustained treatment.^12^ In cancer therapy, for example, conventional chemotherapeutic formulations often suffer from rapid clearance, burst release, insufficient selectivity and systemic toxicity.^13^ Hydrogel systems can address these limitations by improving local drug retention and regulating release kinetics, making them attractive platforms for localized and sustained therapeutic delivery.^14^ These advantages primarily originate from the intrinsic characteristics of hydrogel networks, including their hydrated structure, biocompatibility and cargo encapsulation capability, and are therefore shared across different hydrogel formats. These advantages have positioned hydrogels as important candidates for next-generation biomaterial-assisted treatment strategies.^15^

### 1.2 From bulk hydrogels to hydrogel microspheres

Although conventional bulk hydrogels possess excellent biocompatibility, their clinical translation is often impeded by intrinsic structural limitations. A primary drawback is the requirement for invasive surgical implantation, as pre-formed bulk hydrogels lack the fluidity necessary for minimally invasive delivery. Furthermore, the dense network structure of bulk hydrogels often results in insufficient internal porosity, which restricts cell infiltration and hinders the efficient diffusion of nutrients and metabolic waste, potentially leading to central necrosis in large constructs. Additionally, bulk hydrogels frequently exhibit a mismatch between their macroscopic geometry and the irregular shapes of host tissue defects, compromising interfacial integration. To overcome these challenges, research focus has increasingly shifted toward hydrogel microspheres. Defined as hydrogel particles ranging from approximately 1 to 1000 µm, these microspheres offer superior injectability, modularity, and a high surface-to-volume ratio that enhances mass transport, making them highly adaptable for complex tissue reconstruction.^16^ While hydrogel microspheres retain the intrinsic advantages of hydrogels, including high water content, biocompatibility and cargo encapsulation capability, their microscale architecture provides several additional biomedical advantages that are not readily achievable with conventional bulk hydrogels. Their small size allows administration through syringes or catheters, making them suitable for minimally invasive delivery of drugs, proteins and living cells.^17^

The performance of hydrogel microspheres is determined by the interplay between material chemistry, network architecture and fabrication strategy. Physical and chemical crosslinking play pivotal roles in regulating mechanical stability, degradation behavior, and stimulus responsiveness of polymeric materials. Specifically, the network architecture, whether formed by covalent bonds or physical associations, dictates how macroscopic forces are transduced to the molecular scale. Recent studies on nanostructured block copolymers highlight the significance of physical crosslinking domains in enhancing mechanical robustness and controlling mechanochemical responses. For instance, in a series of poly(methyl methacrylate)-*block*-poly(*n*-butyl acrylate)-*block*-poly(methyl methacrylate) (PMMA-*b*-PnBA-PMMA) triblock copolymers, the self-assembly of glassy PMMA end-blocks into nanoscale domains acts as physical cross-linkers, forming a network that effectively transduces stress to the rubbery PnBA midblock.^18^ This study demonstrated that the composition and morphology of these physically crosslinked networks directly influence the mechanical properties and the onset of mechanochemical activation; increasing the volume fraction of the glassy phase resulted in higher Young's moduli and shifted the activation onset to lower strains but higher stresses. These findings underscore that physical crosslinking through microphase separation is a powerful strategy for tailoring mechanical stability and force-responsive behavior, complementing the structural control offered by fabrication techniques such as emulsion polymerization, electrospraying, microfluidics, and photolithography, which primarily determine particle size, dispersity, shape, throughput, and biological compatibility. In biomedical applications, these parameters directly influence cargo loading, release kinetics, cell viability, scaffold porosity and *in vivo* behaviour. Therefore, a systematic understanding of how hydrogel network design and microsphere fabrication jointly control biological performance is essential for advancing this field.

**Scheme 1:**
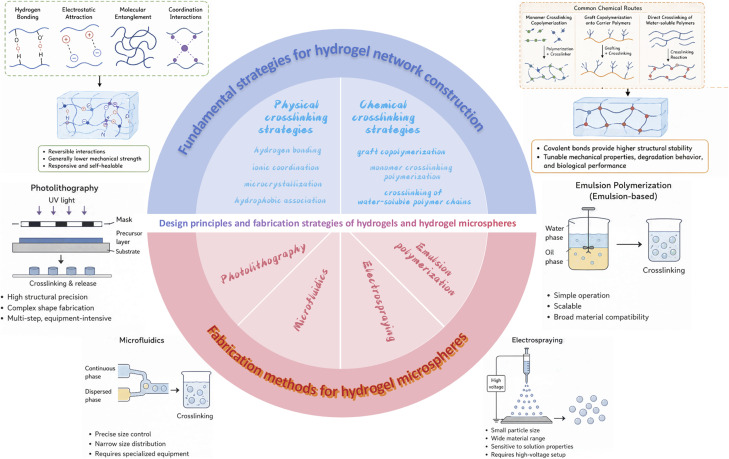
Schematic drawing of hydrogel microspheres for precision biomedicine.

This review covers the integration of the “precision biomedicine” framing and synthesizes emerging literature, we filling critical research gaps by unifying previously isolated sub-disciplines to provide a holistic analysis of hydrogel microspheres (Scheme 1). We first summarize fundamental network construction strategies, emphasizing physical and chemical crosslinking mechanisms, and systematically compare major fabrication routes alongside their respective advantages and limitations. Building on this interdisciplinary foundation, the review examines representative biomedical applications-including drug and cell delivery, tissue engineering, soft tissue filling, neural guidance, and compartmentalized bioactive systems—while revealing previously unrecognized connections that offer novel theoretical frameworks.^15^ Furthermore, through a comprehensive gap analysis, we highlight underexplored areas and discuss the major challenges limiting clinical translation. Beyond identifying these gaps, this work critically examines methodological limitations in existing studies and proposes improved research designs and analytical approaches to enhance the rigor and reproducibility of future investigations.

Compared with previous reviews focusing primarily on fabrication techniques and biomedical applications of hydrogel microparticles, this review places greater emphasis on the structure–property–function relationships governing hydrogel microsphere performance. In particular, we comparatively discuss how network chemistry, particle fabrication strategy, size uniformity, and degradation behavior collectively influence drug delivery efficiency, cell interaction, injectability, and tissue regeneration outcomes. In addition, recent challenges related to large-scale manufacturing, sterilization, storage stability, and clinical translation are critically discussed to provide a more application-oriented perspective for future hydrogel microsphere design. Ultimately, this review serves as both a foundational reference and a catalyst for future innovation, outlining promising directions for developing reproducible, scalable, and biologically instructive platforms that advance the field as a whole.

## 2. Design principles and fabrication strategies of hydrogels and hydrogel microspheres

### 2.1 Fundamental strategies for hydrogel network construction

Hydrogel preparation can be broadly categorized into physical and chemical approaches. Physical crosslinking techniques rely on various intermolecular forces to form hydrogel networks. Hydrogen bonding plays a crucial role in many natural polymer systems, while electrostatic interactions are particularly important in polyelectrolyte hydrogels. Their large surface area and short diffusion distances facilitate cargo exchange and biological interaction, while their surfaces and internal networks can be modified through physical or chemical strategies to introduce bioactivity, responsiveness or targeting capability.^19^ In addition, microspheres can be assembled into granular scaffolds, in which interparticle voids form microporous pathways that promote nutrient transport, cell infiltration and tissue integration. These features make hydrogel microspheres more than miniaturized versions of bulk hydrogels;^20–22^ rather, they represent a distinct class of programmable biomaterial building blocks.

**Fig. 1:**
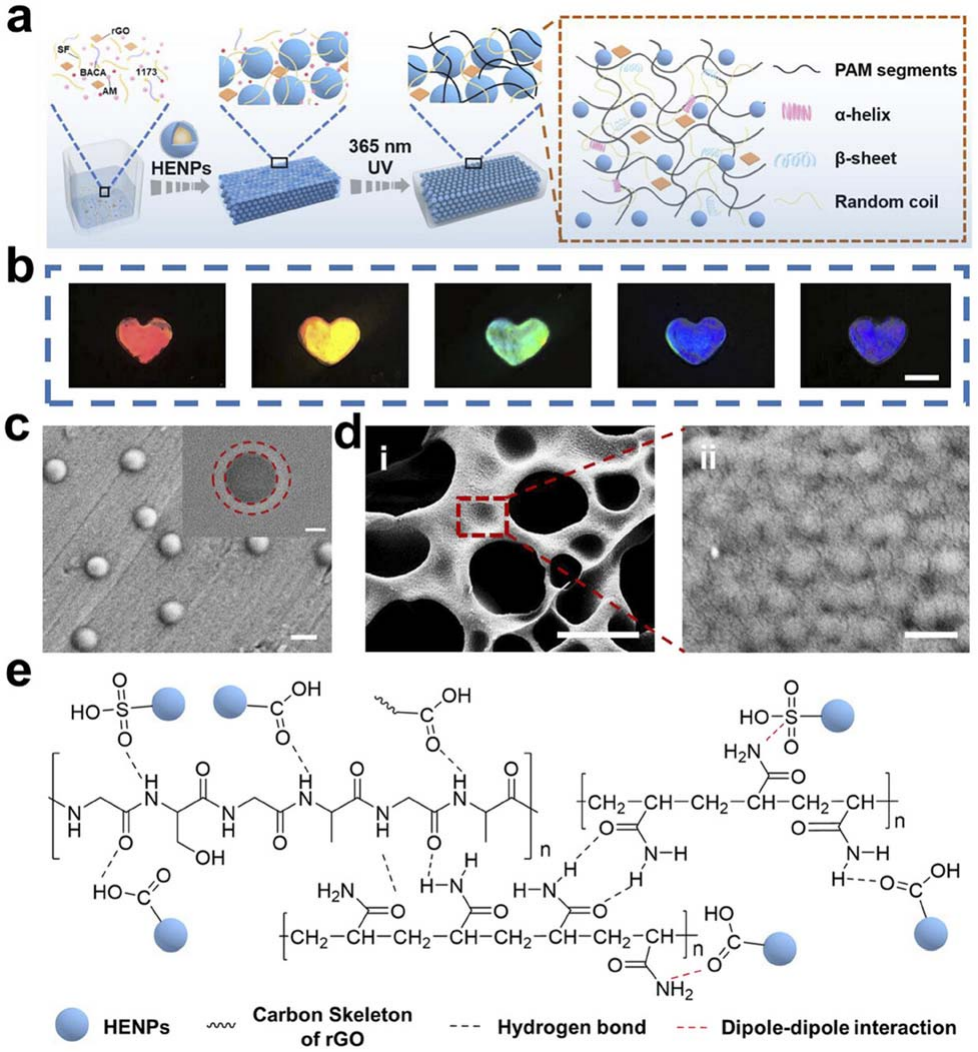
(a) Schematic illustration of the preparation of CSCH. (b) Photographs of CSCH with different structural colors. Scale bar is 1 cm. (c) SEM image of several separated HENPs. Scale bars is 200 nm. Inset on the top-right corner, TEM image of the single HENPs. Scale bars is 50 nm. (d) SEM images of a lyophilized hydrogel sample. Scale bars are 10 µm and 500 nm for (i) and (ii), respectively. (e) Chemical structural formulas of dynamic bonds and dipole–dipole interaction in CSCH. Reproduced with permission from ref. 23 © 2025 The Author(s). Exploration published by Henan University and John Wiley & Sons Australia, Ltd.

Molecular entanglement contributes to the mechanical properties of physically associated networks (Fig. 1), and coordination interactions involving metal ionscan provide additional stability.^23^ Chemical crosslinking involves the formation of covalent bonds between polymer chains, resulting in more permanent and stable hydrogel networks. This approach typically offers better control over network structure and mechanical properties compared to physical methods.^24^ The choice between physical and chemical crosslinking depends on the specific application requirements. Physical methods are often preferred for sensitive biological applications due to their mild conditions and lack of chemical initiators, while chemical methods provide more robust and stable networks suitable for demanding mechanical environments. In practice, these methods allow fine control over mechanical properties, degradation behavior, and biological performance. Frequently used reactions include carbodiimide-mediated coupling, which forms amide linkages between amine and carboxyl groups, and a broad set of click reactions such as copper-catalyzed azide–alkyne cycloaddition, Michael addition, thiol–ene reactions, disulfide formation, and hydrazone or oxime chemistry.^25–29^

#### 2.1.1 Physical crosslinking strategies

Physical methods for hydrogel preparation are diverse and can be mediated through hydrogen bonding, microcrystallization, hydrophobic association, or ionic coordination. For example, Wang *et al.* prepared a tough supramolecular hydrogel through copolymerization of methacrylic acid (MAAc) and methacrylamide (MAAm), in which hydrogen bonding between carboxyl and amide groups played a central role in constructing a stable supramolecular network.^30^ Because these hydrogen bonds are dynamic, the resulting hydrogel not only responded to temperature and deformation rate but also exhibited shape-memory behavior.

Microcrystallization and hydrophobic association have emerged as pivotal physical strategies for reinforcing hydrogel networks, enabling tunable mechanical properties without the need for chemical crosslinkers. Zhang *et al.* successfully fabricated a double-network poly(ethylene glycol)/poly(vinyl alcohol) (PEG/PVA) hydrogel exhibiting high mechanical strength through repeated freeze–thaw cycles.^31^ In this system, PVA formed a rigid percolating skeleton *via* microcrystallization, while PEG chains crystallized within the interstitial spaces, resulting in a hydrogel with a tensile strength of approximately 1 MPa and a Young's modulus of 0.7 MPa. Complementarily, Agrawal *et al.* demonstrated that hydrophobic association significantly enhances the mechanical performance of amphiphilic copolymers, such as poly(ethylene oxide)–poly(lactic acid) (PEO–PLA).^32^ The study revealed that the aggregation of hydrophobic PLA moieties promotes intermolecular association and micellar reinforcement, leading to a substantial increase in the storage modulus. Specifically, the research highlighted that the mechanical robustness is directly correlated with the degree of hydrophobic aggregation, where the formation of physical crosslinks *via* these aggregates effectively dissipates energy under stress. Together, these studies illustrate that physical reinforcement through crystalline domains or hydrophobic microdomains is a versatile approach for designing high-strength hydrogels.

**Fig. 2:**
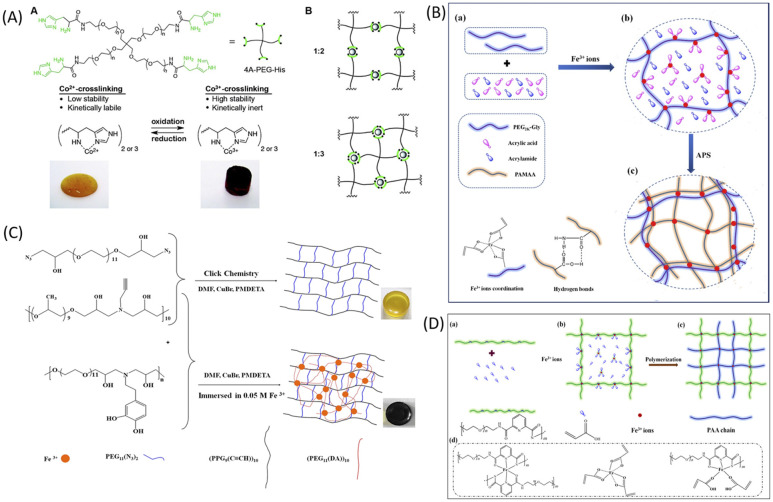
Representative physical and chemical strategies for hydrogel network construction. (A) Ligand bond stability and gel properties of crosslinked networks mediated by Co^2+^ and Co^3+^. Reproduced with permission from ref. 33 Copyright © 2016, American Chemical Society. (B) Final formation of PEG/PAMAA hydrogels by attaching monomers to the PEG template *via* Fe^3+^ ion coordination. Reproduced with permission from ref. 39 © 2018 Elsevier Ltd. (C) Preparation of PEG hydrogels with single and interpenetrating networks through click chemistry combined with Fe^3+^ coordination. Reproduced with permission from ref. 38 Copyright © 2016 John Wiley & Sons, Ltd. (D) Formation of the first network by PEG *via* Fe^3+^ ion coordination, PEG–AA conjugate and free AA monomer subsequently polymerize through Fe^3+^ ion coordination to form the second network, forming an integrated PEG/PAA hydrogel. Reproduced with permission from ref. 36 © 2018 Wiley-VCH Verlag GmbH & Co. KGaA, Weinheim.

Ionic coordination is another common route for physically crosslinked hydrogel formation (Fig. 2). Metal ions such as Ca^2+^, Fe^3+^, and Co^2+^ can interact with polymer chains to establish reversible yet mechanically effective networks.^33–36^ For instance, Ca^2+^-triggered peptide hydrogels have been used for efficient drug encapsulation and controlled release.^37^ Liu *et al.* developed PEG/PAA and PEG/PAMAA double-network hydrogels through Fe^3+^-mediated coordination between polymer chains and monomeric segments, producing materials with high stretchability, self-healing behavior, and improved mechanical performance.^38^ In recent years, hybrid strategies that combine multiple physical or orthogonal interactions have become increasingly important. Liu *et al.*, for example, constructed an interpenetrating network hydrogel in which one network was formed by click chemistry between azide-modified PEG and alkynyl-functional linear PPG derivatives, whereas the second network originated from Fe^3+^ coordination with poly(ethylene glycol-dopamine).^39^

#### 2.1.2 Chemical crosslinking strategies

Chemical hydrogel synthesis can be further divided into monomer crosslinking polymerization, graft copolymerization, and crosslinking of water-soluble polymer chains.^40^ Among these strategies, click chemistry has become one of the most versatile and widely adopted approaches because of its high efficiency, selectivity, and mild reaction conditions. Click reactions generally involve heteroatom-linked bond formation and can include cycloadditions, nucleophilic ring-opening reactions, non-aldehyde carbonyl chemistry, and additions across carbon–carbon multiple bonds,^41^ such as the Diels–Alder (DA) reaction, which has been strategically employed in polymerized ionic liquids (PILs) to achieve stimuli-responsive properties. This chemical strategy allows for the temporary immobilization of ions *via* covalent bonding to the polymer backbone, with the DA reaction specifically facilitating the formation of six-membered rings through cycloaddition between a diene and a dienophile.^42,43^

Representative examples illustrate how click chemistry enables precise network engineering. Ren *et al.* used PEGDA and triethylamine to prepare anti-freezing and heat-resistant ionogels through thiol–ene click crosslinking.^44^ Xin *et al.* employed thiol–ene chemistry to produce PEG microgels bearing residual norbornene groups.^27^ Truong *et al.* prepared crosslinked hydrogels from triblock PEG–PPG–PEG by nucleophilic thiol-yne click chemistry and subsequently integrated them with gelatin hydrogels to obtain swelling-suppressing materials with tunable mechanics.^45^ Li *et al.* synthesized PEG-based composite hydrogels using thermally induced CuAAC chemistry, in which microgels with residual norbornene groups were further crosslinked through a heat-reduced Cu(i) catalyst system.^26^ Qayyum *et al.* fabricated PEG hydrogel microspheres by Michael addition between multi-arm PEG acrylates and dithiol crosslinkers using an electrospray-based process,^46^ whereas Utama *et al.* developed printable bioinks through Michael addition between thiols and maleimides.^47^

Additional chemical systems have been designed to improve biodegradation, responsiveness, and biological performance. Hunckler *et al.* linked PEG-dithiol to amide-connected four-arm PEG-norbornene under light initiation to produce peptide-containing PEG hydrogels with enhanced *in vivo* degradability.^48^ Yu *et al.* prepared PEG hydrogels from four-arm PEG maleimide and peptides through Michael addition and showed, by SEM, rheology, and bioactivity analyses, that the resulting hydrogels had good biocompatibility and enzyme-responsive degradability, supporting their use in tissue engineering.^49^ Sharma *et al.* generated hydrogels from eight-arm PEG aldehyde and eight-arm PEG hydrazide through hydrazone linkages, yielding thixotropic and self-healing materials because of the reversible nature of these bonds.^50^ More broadly, chain-growth polymerization mechanisms and deliberate insertion of functional groups during synthesis strongly influence the final structure and properties of PEG-based hydrogels.^51–54^

### 2.2 Fabrication methods for hydrogel microspheres

A variety of processing methods have been developed for hydrogel microsphere fabrication, among which emulsion polymerization, microfluidics, photolithography, and electrospraying are the most representative.^55–58^ In comparison with microfluidic, lithographic, or electrospray methods that often require more specialized equipment, emulsion-based fabrication remains attractive because of its operational simplicity and scalability.^59^ In most cases, these methods begin with a precursor solution capable of gel formation, followed by physical or chemical crosslinking to solidify droplets into microspheres. Because processing parameters directly affect droplet generation rate, particle size distribution, and crosslinking efficiency, the final properties of microspheres are highly dependent on manufacturing conditions. Therefore, the choice of fabrication route should be matched to the rheological behavior of the precursor solution, the crosslinking mechanism, the desired particle size, and the required dispersibility or structural precision.

#### 2.2.1 Emulsion polymerization

Emulsion polymerization, first proposed in 1932, is among the simplest and most widely used approaches for hydrogel microsphere preparation.^60^ In this method, the dispersed phase containing the hydrogel precursor is mixed with a continuous phase under agitation to form droplets, and surfactants are typically added to stabilize the emulsion. Depending on the formulation, water-in-oil (W/O), oil-in-water (O/W), and even double-emulsion configurations such as W/O/W or O/W/O can be generated. The resulting droplets are then crosslinked, either by adding chemical crosslinkers or by triggering *in situ* photopolymerization, to form hydrogel microspheres. Agitation speed and mixing intensity play a major role in determining particle size and dispersity.

**Fig. 3:**
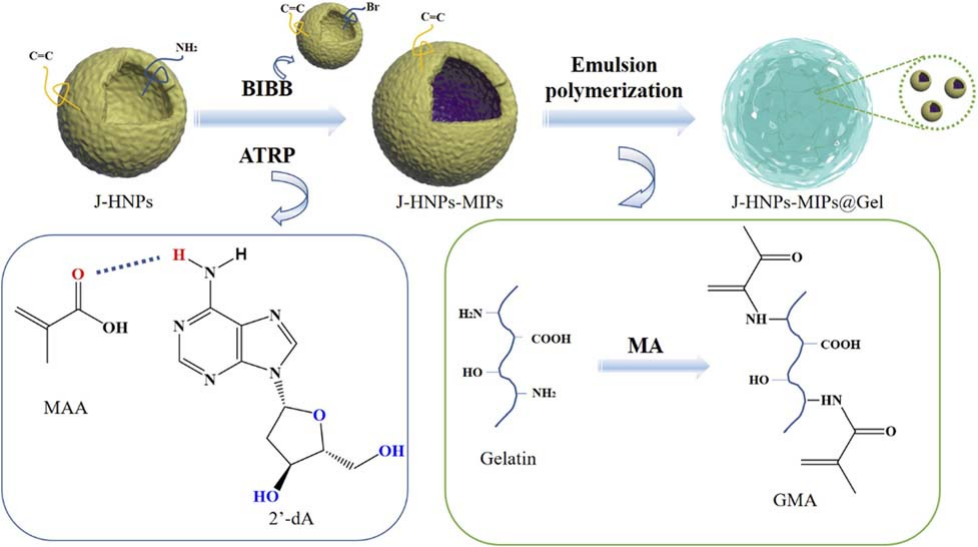
Synthesis route of hybrid hydrogel microspheres loading single-hole hollow imprinted particles. Reproduced with permission from ref. 63 © 2022 Elsevier B.V. All rights reserved.

A major strength of emulsion-based fabrication is its compatibility with bioactive cargoes. Drugs, proteins, or cells can be pre-mixed with the precursor solution before emulsification, enabling the large-scale generation of loaded microspheres.^61,62^ Zhou *et al.*^63^ fabricated hybrid hydrogel microspheres loading single-hole hollow imprinted particles (Fig. 3). In practice, batch emulsification offers high throughput with relatively simple equipment, where production efficiency is primarily limited by vessel volume and mixing quality. This practicality explains why emulsion polymerization remains a common strategy in biomedical hydrogel microsphere preparation. However, a critical limitation identified in these studies is the difficulty in achieving a narrow particle size distribution and the potential shear-induced damage to encapsulated bioactive agents during the high-speed homogenization process. These drawbacks have subsequently triggered extensive research into microfluidic technologies, which provide superior control over droplet generation to ensure high monodispersity and offer a gentler, more precise environment for cell and drug encapsulation.

#### 2.2.2 Electrospraying

Electrospraying is an emerging technique for hydrogel microsphere production in which an electric field is used to disperse a liquid polymer stream into fine droplets or fibers.^64^ When voltage is applied to a flowing polymer solution, charges accumulate within the liquid. Once the electrostatic force exceeds the surface tension at the nozzle, a jet is emitted and undergoes rapid stretching because of charge repulsion. Solvent evaporation or immediate crosslinking then converts the jet into solidified particles. In hydrogel microsphere preparation, particle size is typically governed by applied voltage, needle diameter, and flow rate, and microspheres with diameters approaching 1 µm have been reported.^58^

Despite this advantage in size reduction, achieving strict monodispersity by electrospraying remains challenging. Particle size uniformity varies substantially among different fabrication strategies. Conventional emulsion methods generally produce broader particle size distributions because droplet formation is dominated by bulk shear forces, whereas electrospray systems typically achieve moderate uniformity. In contrast, microfluidic approaches can commonly generate hydrogel microspheres with coefficients of variation (CVs) below 5% owing to their highly controllable droplet formation processes.^53,65,66^ Alginate microspheres are commonly prepared by spraying sodium alginate into CaCl_2_ solution to induce ionic crosslinking.^67^ Similarly, chitosan-based precursors can be electrosprayed into tripolyphosphate-containing solutions to trigger electrostatic gelation.^68^ Importantly, electrospraying is also compatible with biological applications and has been used for cell encapsulation in hydrogel microspheres.

#### 2.2.3 Microfluidics

Microfluidic technology has advanced rapidly and now provides highly controllable routes for generating microscale droplets within confined channels. In the field of hydrogel microspheres, microfluidics allows not only precise size control but also the incorporation of functional materials and structural heterogeneity, thereby enabling stronger integration with pharmacology, regenerative medicine, and bioengineering.

**Fig. 4:**
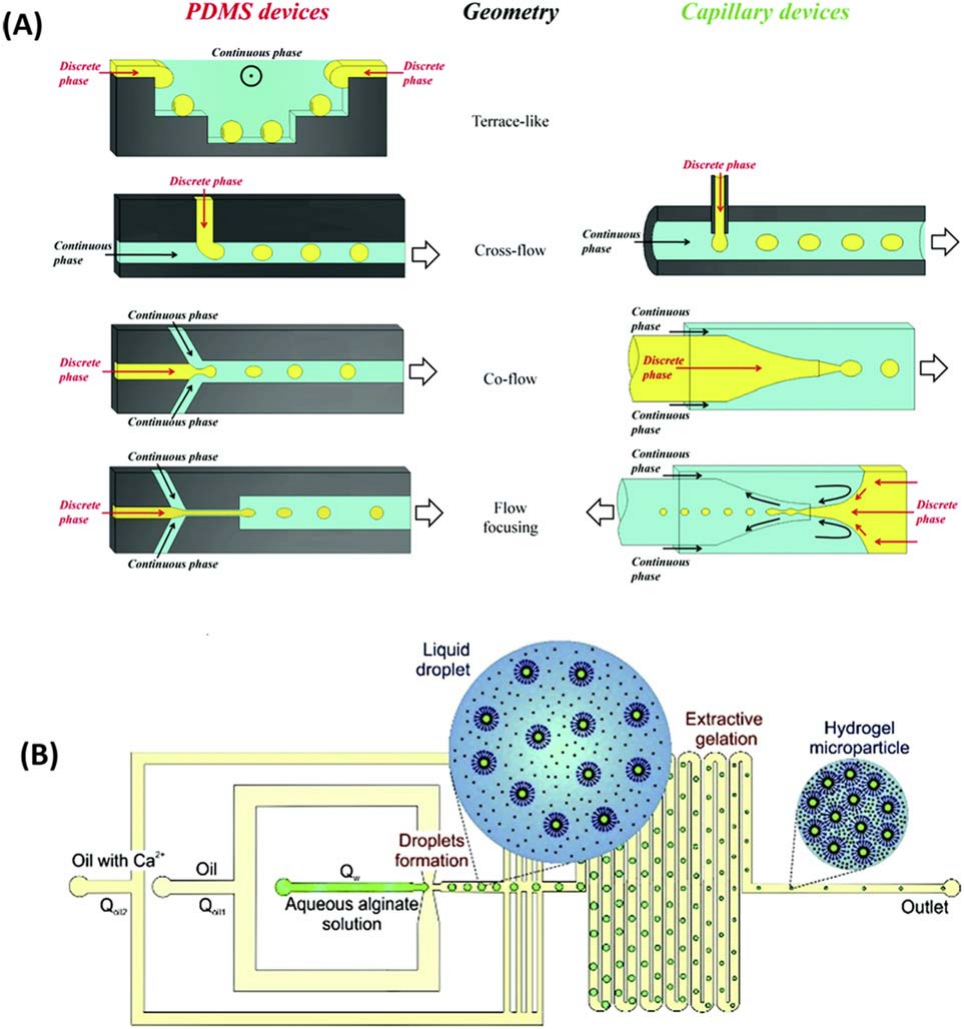
(A) Representative PDMS-based and capillary-based microfluidic devices for hydrogel microsphere generation. Reproduced with permission from ref. 66 2019 published by Royal Society of Chemistry. (B) Composite microparticles and their orientation in the magnetic field. Reproduced with permission from ref. 69 2016 published by Royal Society of Chemistry.

The concept of droplet-based microfluidics was introduced by Thorsen *et al.* in 2001,^70^ and subsequent studies by Nie and others demonstrated its suitability for biomedical applications because microspheres fabricated in this way can exhibit favorable biocompatibility and accommodate biological cargoes.^71,72^ Current devices are commonly categorized into polydimethylsiloxane (PDMS)-based systems and capillary-based systems.^66^ In PDMS devices, the dispersed and continuous phases meet within a patterned channel and droplet breakup occurs through shear at the junction (Fig. 4). In capillary systems, the two phases flow through separate tubes and meet at the interface, where the dispersed phase is sheared into droplets by the continuous phase. Cross-flow, co-flow, and flow-focusing geometries are widely used variants of these principles.

At the mechanistic level, microfluidic hydrogel microsphere fabrication depends on controlling droplet formation at channel intersections, where shear forces and interfacial interactions promote the generation of aqueous droplets within an oil phase. By adjusting channel geometry and the relative flow rates of the two phases, microsphere diameters can typically be tuned within the range of 5–500 µm.^69,73^ However, despite the excellent control over size and morphology, microfluidics still faces several limitations, including strict operating conditions, limited throughput, sensitivity to precursor rheology, and incomplete understanding of multiphase transport and mass transfer within narrow channels.

#### 2.2.4 Photolithography

Photolithographic fabrication of hydrogel microspheres mainly relies on light-triggered polymerization within miniaturized templates or spatially defined exposure regions. Analogous to this spatially controlled polymerization approach, recent advancements in solid polymer electrolytes have demonstrated a “locking” mechanism for configuring electronic junctions, which can be conceptually extended to the fabrication of functional microstructures. Specifically, the use of a custom-synthesized, doubly-polymerizable ionic liquid (DPIL) enables the immobilization of ions through a triggered polymerization process. In this method, ions are first positioned under an electric field to form specific doping profiles, and then locked in place *via* a thermal curing process that solidifies the electrolyte matrix. This results in stable, reconfigurable patterns of charge distribution.^74^ In parallel, by controlling the geometry of photomasks or hydrogel molds, photolithography enables regulation of microsphere shape, size, and dispersity.^75^ Because this method does not necessarily require surfactants or oil phases, it is especially attractive for cell encapsulation and other biologically sensitive applications. Broadly, photolithographic routes can be divided into imprint lithography, conventional photolithography, and stop-flow or flow lithography.^76^ Imprint lithography fills a mold with precursor solution and solidifies the particles after polymerization. Conventional photolithography uses patterned light shielding to selectively cure defined regions, whereas flow lithography periodically exposes a moving precursor stream to produce microspheres *in situ*.

Advances in microfabrication have enabled the generation of smaller and more sophisticated templates, making it increasingly feasible to design hydrogel microspheres with tailored external shapes and internal architectures.^75,77,78^ Nevertheless, some geometric constraints remain. In imprint lithography, for example, excessively complex three-dimensional structures can be difficult to remove intact from the mold after crosslinking. As a result, commonly fabricated shapes still include relatively simple cubes, circles, rods, and cuboids.^57^ To overcome these limitations, multiphoton light sources and sequential polymerization strategies have been explored for the fabrication of more complex three-dimensional hydrogel particles.^78–80^ Acrylated PEG is among the most common materials used in photolithographic hydrogel microsphere fabrication because of its tunable properties and strong cell compatibility, and natural polymers such as hyaluronic acid and gelatin can also be functionalized for light-mediated microsphere production.^81,82^

Overall, emulsion-based methods remain attractive for scalable manufacturing but usually sacrifice particle uniformity. Electrospray approaches provide better control over particle morphology but are limited by nozzle instability and relatively low throughput. Microfluidic technologies offer excellent monodispersity and cargo preservation, although large-scale production remains technically challenging. Lithographic methods provide precise structural control but often require complex instrumentation and higher production costs. Therefore, selecting an appropriate fabrication route requires balancing scalability, particle uniformity, biological compatibility, and application-specific functional requirements.

**Scheme 2:**
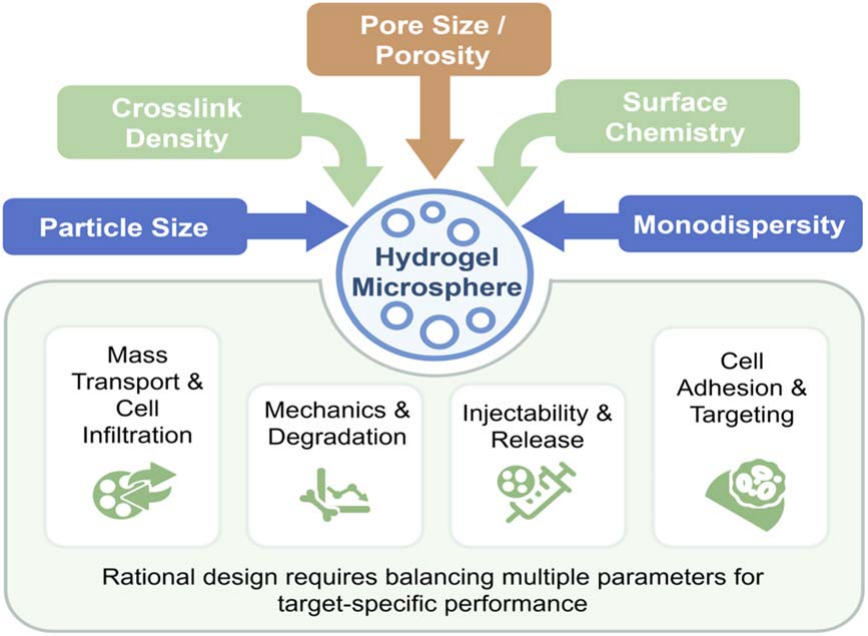
Design–function relationships in hydrogel microspheres.

The interplay between network chemistry, particle geometry, fabrication precision, and microsphere assembly directly influences cargo loading, release behavior, cell compatibility, mechanical performance, and *in vivo* functionality. These design–function relationships are schematically summarized in Scheme 2. Table 1 summarizes the major fabrication methods for hydrogel microspheres, including their typical particle size range, dispersity, throughput, cell compatibility, equipment complexity, and representative polymer systems.

**Table 1:** Summary table: fabrication methods for hydrogel microspheres

Method	Principle & mechanism	Key characteristics & materials	Advantages	Limitations	Ref.
Emulsion polymerization	Dispersed phase mixed with continuous phase under agitation to form droplets (W/O, O/W), stabilized by surfactants, followed by crosslinking	Control: particle size determined by agitation speed/mixing intensity	Simplicity: operationally simple and scalable	Uniformity: difficult to achieve narrow particle size distribution	60–63
		Materials: compatible with various precursors	Capacity: high throughput; compatible with bioactive cargoes (drugs, cells)	Safety: high-speed homogenization may cause shear-induced damage to bioactive agents	
Electrospraying	Electric field disperses polymer liquid into fine droplets/jets. Charges accumulate, overcoming surface tension; solvent evaporation or crosslinking solidifies particles	Control: size governed by voltage, needle diameter, and flow rate	Miniaturization: capable of producing very small particles (approaching 1 µm)	Uniformity: achieving strict monodispersity is challenging (CV often >5%)	53, 54 and 65–68
		Materials: alginate (with CaCl_2_), chitosan (with tripolyphosphate)	Application: compatible with cell encapsulation	Complexity: requires precise control of electrical parameters	
Microfluidics	Precise control of droplet formation in confined channels (PDMS or capillary-based) *via* shear forces at channel intersections (cross-flow, co-flow, flow-focusing)	Control: diameter tunable (5–500 µm) *via* channel geometry and flow rates	Precision: excellent control over size, morphology, and structural heterogeneity	Throughput: limited production volume	66, 69 and 71–73
		Materials: highly versatile for functional materials	Quality: high monodispersity; gentle environment for biological cargoes	Constraints: strict operating conditions; sensitivity to precursor rheology; complex multiphase transport	
Photolithography	Light-triggered polymerization within miniaturized templates or spatially defined exposure regions (imprint, conventional, or flow lithography)	Control: shape/size defined by photomasks or molds	Biocompatibility: often surfactant/oil-free, ideal for sensitive cells	Geometry: complex 3D structures can be difficult to remove from molds	57 and 74–82
		Materials: acrylated PEG, functionalized hyaluronic acid, gelatin	Design: capable of tailored external shapes and internal architectures	Shape: often limited to simple shapes (cubes, rods) unless using advanced multiphoton sources	

## 3. Biomedical applications of hydrogel microspheres

### 3.1 Functional advantages and overall performance

Hydrogel microspheres combine the hydrated three-dimensional architecture of hydrogels with the geometric and interfacial benefits of particulate systems. As a result, they offer high specific surface area, tunable porosity, facile functionalization, and compatibility with drugs, biomacromolecules, and cells. These features make hydrogel microspheres particularly suitable as carriers for therapeutic agents and living cells. In many biomedical contexts, they are more versatile than monolithic hydrogels because they are injectable, can be assembled into porous scaffolds, and permit modular combinations of multiple microsphere populations with different functions or degradation profiles.

### 3.2 Drug delivery

For drug delivery, the highly porous structure of hydrogels provides a favorable microenvironment for cargo loading, whereas the microsphere format further increases surface area and decreases transport distance. Compared with bulk hydrogels, microsphere-based systems can be injected through needles or catheters and delivered into a broader range of tissues. They also avoid some of the practical limitations of bulk gels, such as the need for *in situ* gelation or shear-induced fragmentation at the application site. Because multiple microsphere populations can be co-administered, the platform is also well suited for combination delivery or for generating distinct release profiles within one treatment.

**Fig. 5:**
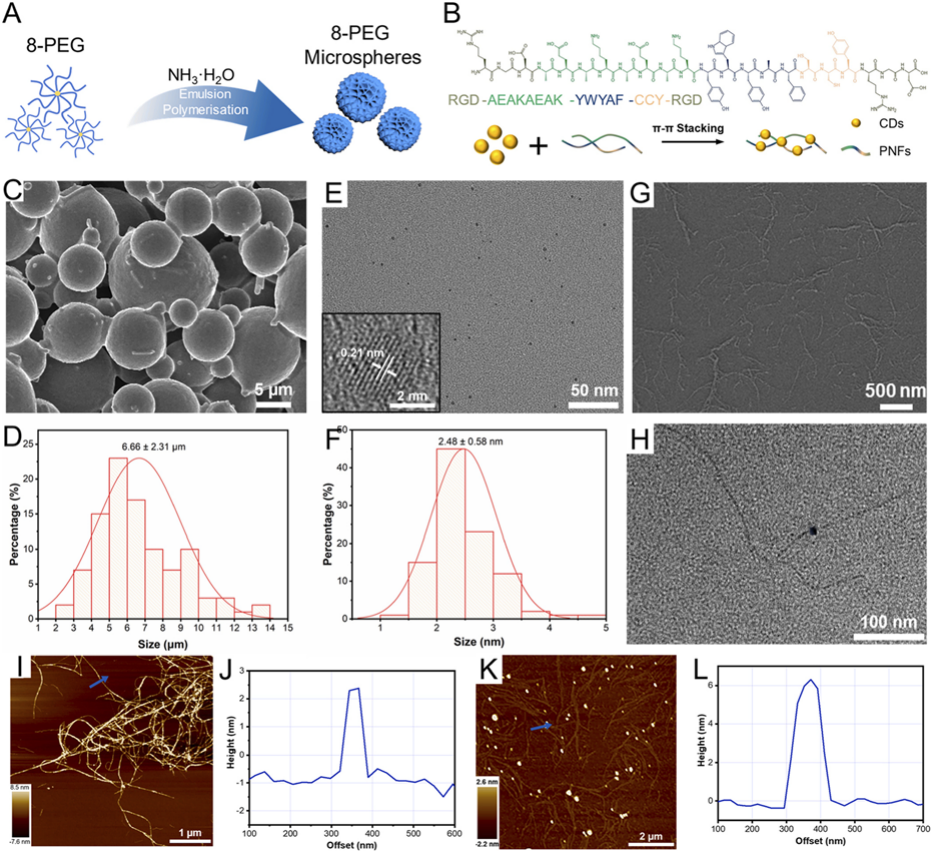
Morphological analysis of hydrogel microsphere components. (A) The formation mechanism of hydrogel microspheres. (B) Sequence diagram of PNFs and formation mechanism of PNFs-CDs. (C and D) SEM image of PEG hydrogel microspheres and the corresponding size distribution. (E and F) TEM image of CDs and the size distribution. (G) SEM image of PNFs. (H) TEM image of PNFs-CDs. (I–L) AFM images and the height distributions of PNFs and PNFs-CDs. Reproduced with permission from ref. 83 © 2024 published by Elsevier Ltd.

Xiao *et al.*^83^ reports an all-in-one hydrogel drug carrier, DOX@PEG/PNFs-CDs, constructed from doxorubicin-loaded PEG hydrogel microspheres and metformin-derived carbon-dot-loaded peptide nanowires (Fig. 5). The porous PEG hydrogel microspheres exhibited a porosity of 82.5 ± 3.2%, enabling high drug loading efficiency of 92.7 ± 4.1% for DOX, good biodegradability with 85% degradation after 14 days, and pH-responsive drug release, with faster DOX release under weakly acidic tumor-like conditions (pH 5.5) showing a cumulative release of 78.4 ± 5.3% at 72 hours compared to 42.1 ± 3.8% at physiological pH (7.4). The RGD-containing peptide nanowires endowed the carrier with specific targeting ability toward αvβ3-overexpressing MDA-MB-231 breast cancer cells. By combining the chemotherapeutic effect of DOX with the cytotoxic contribution of metformin-derived carbon dots, the system showed synergistic inhibition of breast cancer cell growth. Overall, the study presents a degradable, biocompatible, targetable hydrogel microsphere platform with potential for smart and localized breast cancer therapy.

**Fig. 6:**
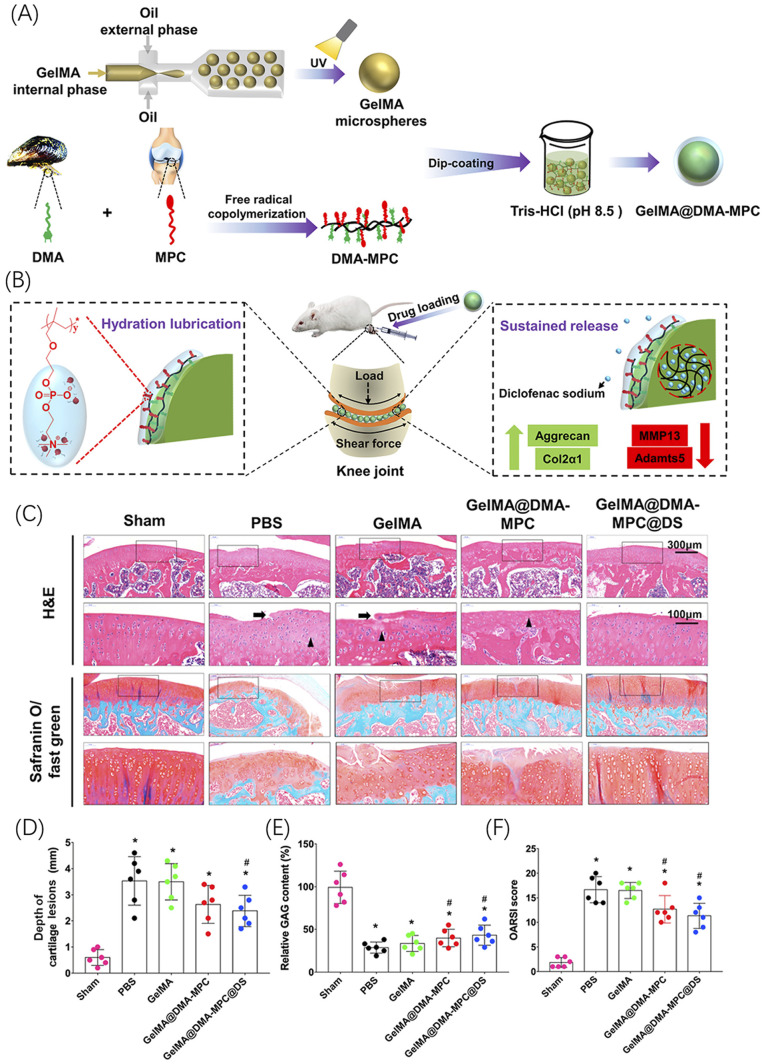
GelMA@DMA–MPC composite hydrogel microspheres for drug delivery. (A) Preparation process of GelMA@DMA–MPC composite hydrogel microspheres. (B) Therapeutic mechanism of drug-loaded GelMA@DMA–MPC microspheres. (C) Representative images of H&E staining and safranin O-fast green staining in the histological staining assay display the histological changes in various degrees of cartilage section for different groups of rats at 8 weeks after the DMM surgery. The black arrow shows erosion fissures, and the black triangle represents tissue cellularity with cloning. (D) The corresponding depth of cartilage lesions, (E) relative GAG content and (F) OARSI score of articular cartilage in different groups of rats. Reproduced with permission from ref. 85 © 2021 The Authors. Publishing services by Elsevier B.V. on behalf of KeAi Communications Co. Ltd.

Consistent with these advantages, hydrogel microspheres have been widely investigated for therapeutic delivery. Lai *et al.* used a microfluidic electrospray strategy to prepare homogeneous core–shell microspheres for drug delivery and tuned particle size by varying electric field strength, flow rate, and polymer concentration while using methylene blue and tetracycline hydrochloride as model drugs.^84^ Han *et al.* developed lubricating and drug-loaded hydrogel microspheres for osteoarthritis treatment by preparing GelMA microspheres *via* microfluidics and coating them with a DMA–MPC biomimetic lubricating layer (Fig. 6), followed by diclofenac sodium loading to achieve injectable controlled anti-inflammatory therapy.^85^ In tumor therapy, Dong *et al.* designed near-infrared-responsive hydrogel microspheres capable of sensing pH changes in the tumor microenvironment while simultaneously carrying antitumor agents, thus integrating monitoring and treatment.^86^

Hydrogel microspheres have also shown promise in oral delivery systems. Insulin delivery is a representative example, because hydrogels can protect insulin in the acidic stomach environment and release it in the intestine, where absorption is more favorable.^87^ Microsphere suspensions provide additional advantages because their small size facilitates intestinal transit and local interaction with the mucosa. Considerable effort has therefore been directed toward pH-responsive microspheres that remain stable in the stomach but release insulin in the small intestine. Polymers containing free carboxylic acid groups, such as poly(methacrylic acid), are particularly useful because they undergo protonation and deprotonation in response to pH, thereby altering swelling and degradation behavior.^88–90^

To prolong residence time, mucoadhesive components such as chitosan can be introduced. Other designs exploit glucose-responsive behavior for self-regulated insulin administration. For example, Gu *et al.* developed electrosprayed chitosan-based microgels integrating insulin with glucose-sensitive enzyme nanocapsules. Under hyperglycemic conditions, glucose oxidase catalyzed the conversion of glucose into gluconic acid, leading to protonation and swelling of the chitosan network and thereby accelerating insulin release. This closed-loop delivery strategy demonstrated glucose-dependent insulin administration and highlighted the potential of hydrogel microspheres for intelligent diabetes management.^68^

The interaction between drugs and the hydrogel matrix is also critical for sustained release. Covalent, electrostatic, and hydrophobic interactions can all strengthen cargo retention and improve release control.^91,92^ Heparin, because of its high sulfation level and affinity for proteins, has been widely incorporated into hydrogel microspheres to stabilize and deliver growth factors such as BMP2, FGF2, and VEGF. Moreover, because microspheres with different degradation rates can be mixed prior to injection, composite formulations can create more selective local biological environments. Mealy *et al.*, for example, prepared injectable mixtures of stable and degradable hyaluronic acid particles by microfluidics and demonstrated that biphasic microsphere systems showed faster cargo release and more rapid degradation than single-component systems.^93^

### 3.3 Cell delivery

The delivery of viable cells to damaged or diseased tissues provides important therapeutic opportunities, but major challenges remain, including low survival after transplantation and poor integration with host tissue. To address these problems, researchers have increasingly explored biomaterial carriers that can improve cell retention, survival, and functional performance.^94^ Hydrogels are especially attractive for this purpose because they can improve cell localization after injection and provide a supportive physicochemical microenvironment that facilitates cell survival and integration.^95,96^ However, the limited porosity and poor injectability of many bulk hydrogels have restricted their utility. These limitations have, in turn, promoted the use of hydrogel microspheres in cell delivery applications.

Hydrogel microspheres can protect encapsulated cells from mechanical damage and reduce the detrimental effects of shear stress that are often encountered during processing or administration. Hamilton *et al.* developed degradable hyaluronic-acid-based microcapsules for stem cell encapsulation and delivery, using a core–shell fabrication strategy to obtain highly cytocompatible acrylic hyaluronic acid microspheres.^97^ In endodontic regeneration, injectable microspheres are particularly appealing because the root canal system is structurally complex and irregular. Zhang *et al.* prepared RGD-modified alginate/LAPONITE^®^ hydrogel microspheres with diameters of about 400 µm and encapsulated human dental pulp stem cells together with VEGF. *In vivo* tests showed clear growth of pulp-like tissue and neovasculature one month after implantation in a nude mouse model.^98^ The sustained release of VEGF and the presence of LAPONITE^®^ promoted stem cell differentiation *in vitro* and tissue regeneration *in vivo*.

**Fig. 7:**
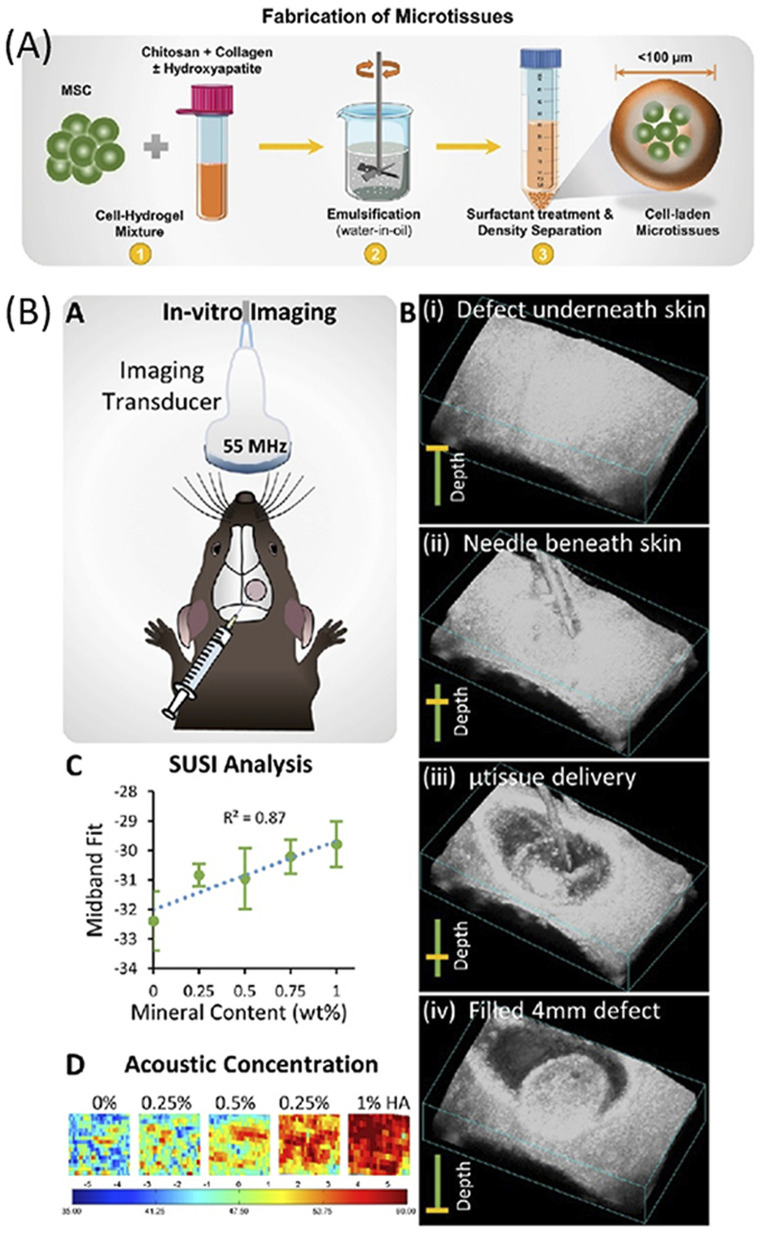
(A) Schematic illustration of hydrogel microspheres loaded with BMSCs. (B) Schematic of the monitoring microtissue implantation into the calvarial defect. A. *In vitro* imaging; B. high-resolution ultrasound imaging; C. SUSI ananysis; D. acoustic concentration. Reproduced with permission from ref. 100 © 2019 Elsevier Ltd.

Bone regeneration is another active application area. Zhao *et al.* used microfluidics to encapsulate bone marrow mesenchymal stem cells (BMSCs) and growth factors in gelatin microspheres, generating injectable osteogenic microtissues in which cells proliferated inside the microspheres and subsequently migrated outward after implantation to guide bone repair.^99^ Similarly, Annamalai *et al.* employed emulsion polymerization to embed BMSCs in chitosan/collagen-based hydrogel microspheres and constructed spheroidal microtissues for bone healing.^100^ In their study, cells were osteogenically pre-differentiated before injection into mouse calvarial defects, and the preconditioned microsphere system improved defect healing compared with undifferentiated controls, likely through beneficial interactions with macrophages after transplantation (Fig. 7).

Hydrogel microspheres have also shown utility in cardiac repair. Cha *et al.* coated gelatin microspheres with a silica hydrogel shell to create a core–shell carrier for culturing cardiac side population cells and demonstrated that the silica layer enhanced cell protection and survival while supporting adhesion and proliferation on the microsphere surface.^101^ Feyen *et al.* demonstrated that the delivery of cardiovascular progenitor cells (CPCs) encapsulated in gelatin hydrogel microspheres significantly enhances therapeutic outcomes in ischemic myocardium compared to the administration of free cells. Specifically, the use of these microspheres—characterized by a porosity of approximately 90%—resulted in a roughly two-fold increase in cell engraftment and significantly improved cardiac function, as evidenced by a marked attenuation in the decline of fractional shortening.^102^

### 3.4 Tissue engineering

Hydrogels have long been investigated as tissue engineering scaffolds for the regeneration and repair of damaged tissues.^103^ In these applications, scaffolds are expected to support cell viability, facilitate adhesion and remodeling, and in some cases provide biochemical cues such as growth factors or chemotactic signals that synchronize tissue formation with repair dynamics. Quantitative studies have demonstrated that microspheres pack into structures with defined interstitial voids, creating microporous pathways with pore sizes typically ranging from 20–100 µm, which facilitate cell infiltration and nutrient diffusion. The porosity of these granular scaffolds can be precisely controlled between 60–90% by adjusting microsphere size and packing density. Mechanical stability analysis reveals that granular hydrogel scaffolds exhibit tunable mechanical properties, with compressive moduli ranging from 5 kPa to 500 kPa depending on the polymer concentration and cross linking density. This range encompasses the mechanical properties of various native tissues, including brain tissue (0.1–1 kPa), adipose tissue (2–10 kPa), and cartilage (100–1000 kPa). In addition, the modularity of microsphere systems allows for precise tuning of mechanical properties, porosity, and cell-interactive ligands. For example, RGD peptide concentration can be controlled from 0.1–1.0 mM to optimize cell adhesion, while growth factor delivery can be engineered to provide sustained release over periods of 7–28 days. The ability to independently control these parameters makes granular hydrogel scaffolds particularly attractive for complex tissue engineering applications.

**Fig. 8:**
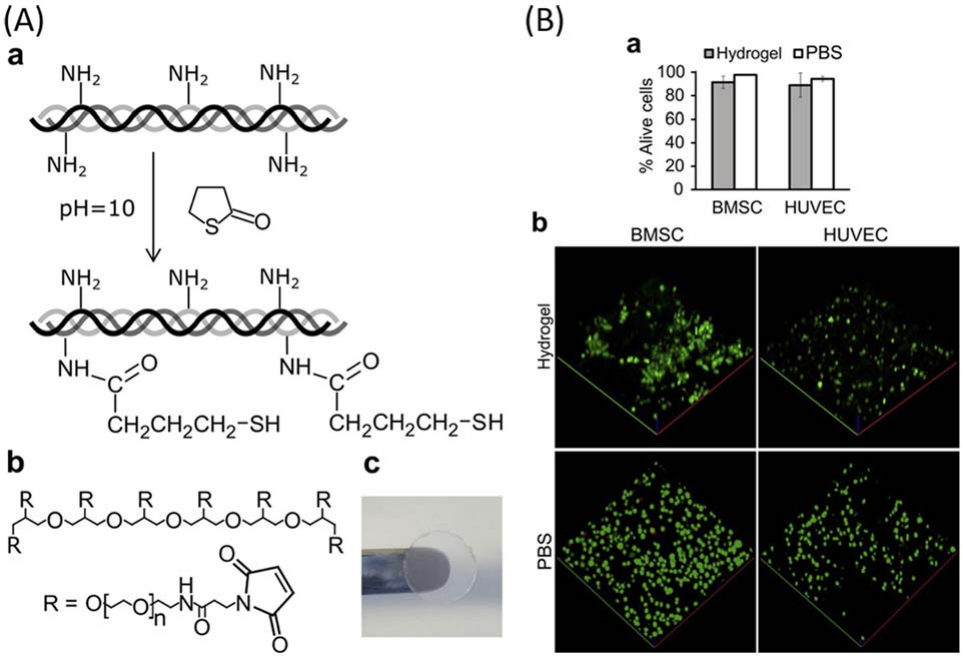
(A) (a) Thiol-collagen synthesis scheme; (b) 8-arm PEG-maleimide used in this study; (c) photograph of demoulded hydrogel. (B) Cell viability 1 h after injection through a 27-gauge needle using thiol-collagen hydrogel or PBS as carriers, assessed using live/dead assay. Reproduced with permission from ref. 25 Copyright © 2019, American Chemical Society.

Early investigations into microsphere-based scaffolds prioritized applications in bone and cartilage regeneration. A representative study by Xin *et al.* demonstrated the fabrication of osteogenic scaffolds through the annealing of gelatinated, millimeter-scale chitosan microspheres. This thermal treatment induced inter-particle fusion, yielding a highly porous 3D architecture with a porosity of approximately 82% and an average pore size of 200 µm. Furthermore, the resulting scaffolds exhibited a compressive modulus of roughly 40 kPa, a mechanical property that not only supports structural integrity but also facilitates the viability and proliferation of encapsulated human mesenchymal stem cells (hMSCs).^104^ Later studies broadened the scope of these systems for accelerated tissue regeneration. For example, PEG-based hydrogel microspheres prepared by microfluidics were injected into skin wounds and annealed through factor XIIIa-mediated amide bond formation between transglutaminase peptide substrates (Fig. 8), producing a stable scaffold that markedly accelerated skin regeneration and even enabled the reappearance of hair follicles after several days.^25^

Microsphere-based scaffolds have also been adapted for spinal cord repair. Truong *et al.* fabricated tubular PEG hydrogel microsphere scaffolds through emulsion-based shaping followed by covalent annealing, producing hollow channel-like architectures that could guide axonal extension.^105^ After implantation into mouse spinal cord injury sites, stacked tubular scaffolds reduced scar formation more effectively than bulk hydrogel controls. In another recent study, Kucharska *et al.* prepared heparin-modified gelatin hydrogel microspheres by batch emulsion polymerization for mandibular bone repair in diabetic rats.^22^ By loading interleukin-4 into the microspheres, the authors exploited heparin-growth factor binding to regulate macrophage-mediated immune responses, reduce inflammation, and enhance osteogenic differentiation. Collectively, these studies demonstrate that microsphere-based scaffolds are not only structural supports but also active regulators of cell behavior and tissue repair.

### 3.5 Emerging and interdisciplinary applications

The biomedical utility of hydrogel microspheres extends beyond the three major areas discussed above. Over the past decade, hydrogel microspheres, especially those based on hyaluronic acid, have been widely used as soft tissue fillers in aesthetic and reconstructive medicine because of their injectability, swelling capacity, and mechanical resemblance to soft tissues.^106^ In such applications, bulk hydrogels are often mechanically processed into microparticulate fillers and mixed with injectable vehicles to facilitate administration and improve *in situ* retention. Microsphere size can be adjusted to match clinical goals, with smaller particles often preferred for wrinkle or scar correction and larger ones used for deeper soft tissue augmentation.

In ophthalmology, recent advancements in hydrogel-based therapies highlight the efficacy of combining biomaterials with cell-free regenerative strategies. Specifically, thermosensitive chitosan-based hydrogels loaded with exosomes derived from induced pluripotent stem cell-mesenchymal stem cells (iPSC-MSC-exos) have proven effective in treating corneal injuries. These exosomes promote repair and reduce scarring by downregulating collagen expression *via* the miR-432-5p/TRAM2 pathway.^107^ Expanding on this, 3D-cultured MSC exosome-functionalized GelMA hydrogels offer a multidimensional approach to heal complex corneal defects involving limbal stem cell deficiency (LSCD) by mitigating inflammation through the miR-150-5p/PDCD4 axis.^108^ Complementing these regenerative approaches, thermoresponsive antioxidant metal-free carbon nanodot (CD) hydrogels have been developed to treat dry eye disease (DED) by scavenging reactive oxygen species (ROS) and reducing apoptosis on the ocular surface.^109^

Hydrogels have also found significant applications in precision medicine, particularly in theranostic systems that integrate diagnostic imaging with therapy. Recent studies highlight their versatility as multifunctional platforms. For instance, microfluidic Gd^3+^-chelated hydrogel microspheres enable MRI-visible, image-guided photothermal chemotherapy for pancreatic cancer, allowing non-invasive tracking of tumor regression.^110^ Concurrently, programmatically activated DNA hydrogel microcapsules (HAMs) have been developed for inflammatory bowel disease (IBD). These HAMs utilize a multi-stage response mechanism to the inflammatory microenvironment, ensuring precise drug release and enhanced local concentration for effective oral therapy.^111^ Together, these innovations demonstrate how hydrogels bridge diagnostic visualization and targeted treatment.

Hydrogel microspheres have also been explored in neuroengineering and bioinspired compartmentalized systems. To guide the linear and directional growth of neural cells, researchers have developed PEG acrylate hydrogel microspheres containing superparamagnetic particles, allowing ordered alignment under an external magnetic field.^112^ Such aligned hydrogel microsphere systems provide anisotropic microenvironments that can promote directional neurite extension and guide axonal regeneration. Compared with conventional bulk hydrogels, aligned microsphere assemblies exhibit improved structural adaptability and enhanced cellular interaction because interparticle voids facilitate nutrient diffusion and cell migration. In spinal cord injury and peripheral nerve repair, directional guidance cues are particularly important for restoring neural connectivity and reducing random axonal growth.

Recent studies have further demonstrated that magnetically responsive or structurally aligned hydrogel microspheres can regulate neural stem cell behavior, improve neurite elongation, and support tissue integration after implantation. In addition to magnetic alignment, microsphere topology, scaffold porosity, and biochemical functionalization may all contribute to neural regeneration efficiency. These findings suggest that hydrogel microspheres may provide promising building blocks for future neural tissue engineering and neuroregenerative medicine. Other studies have designed PEG hydrogel microspheres with multiple compartments that mimic aspects of intracellular organization. These compartmentalized microspheres provide spatially separated microreactors in which different enzymes can be immobilized, enabling pH-specific reactions and controlled transfer of reactants between compartments. Such work highlights the potential of hydrogel microspheres as intelligent and multifunctional biomaterial platforms. To facilitate cross-study comparison, representative biomedical applications of hydrogel microspheres are summarized in Table 2.

**Table 2:** Summary of hydrogel microspheres in biomedical applications

Category	Key advantages	Representative applications	Notable examples	Ref.
Overall performance	High surface area, tunable porosity, easy functionalization, excellent biocompatibility, injectability	Modular assembly into porous scaffolds, combination of different functional microspheres	Combines 3D hydrogel structure with geometric advantages of particle systems	27 and 47
Drug delivery	Enhanced drug loading capacity, reduced mass transfer distance, pH-responsive release	Cancer therapy, oral delivery, combination therapy	DOX@PEG/PNFs-CDs microspheres (82.5% porosity, pH-responsive); P(MAA) microspheres for oral insulin delivery	68 and 83–93
Cell delivery	Protection from mechanical damage and shear stress, improved cell retention and survival	Dental pulp regeneration, bone repair, cardiac repair	RGD-modified alginate/laponite microspheres for dental stem cells; gelatin microspheres for BMSCs in bone regeneration	94–102
Tissue engineering	Controllable porosity (60–90%), tunable mechanical properties (5–500 kPa compressive modulus), mimics natural tissue stiffness	Skin repair, spinal cord regeneration, immune modulation	PEG microspheres for skin wound healing; heparin-modified gelatin microspheres for mandibular repair in diabetic rats	22, 25 and 103–105
Emerging applications	Multifunctional capabilities, precision targeting, stimuli-responsive properties	Cosmetic/aesthetic, ophthalmology, precision medicine, neuroengineering	Hyaluronic acid microspheres as soft tissue fillers; chitosan-based thermosensitive hydrogels for corneal repair; Gd^3+^-chelated microspheres for MRI-visible pancreatic cancer therapy	106–112

## 4. Current challenges and future perspectives

### 4.1 Technical challenges

**Scheme 3:**
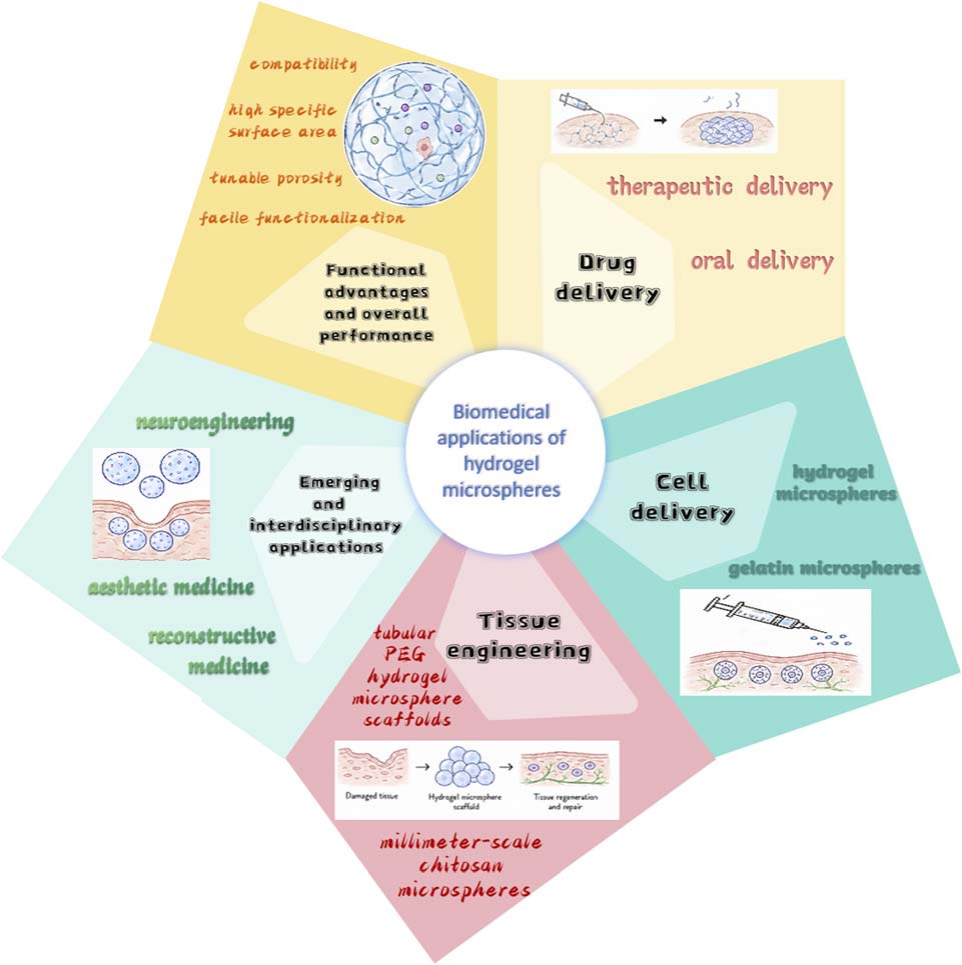
Biomedical applications, clinical translation challenges, and future perspectives of hydrogel microspheres.

Despite considerable advances in hydrogel microsphere design and fabrication, several technical challenges continue to hinder their broader biomedical application and clinical translation. One of the most significant obstacles is the lack of standardized and scalable manufacturing processes. Although microfluidic technologies can generate highly monodisperse hydrogel microspheres with precise control over particle size, morphology, and compartmentalized structures, their production throughput remains substantially lower than that of conventional emulsification methods. This limitation restricts large-scale manufacturing and increases production costs, thereby impeding commercial implementation (Scheme 3).

Another major challenge lies in preserving the biological functionality of encapsulated therapeutic cargos. For cell-laden hydrogel microspheres, maintaining cell viability, proliferation capacity, and phenotype stability throughout fabrication, storage, and implantation remains particularly demanding. For example, Truong *et al.* demonstrated that microporous annealed particle (MAP) hydrogels can effectively support cell spreading, proliferation, and gene transfer through tunable stiffness, pore size, and adhesive properties. However, translating these favorable biological outcomes into reproducible large-scale production remains challenging due to batch-to-batch variability and the sensitivity of living cells to processing conditions.^105^

Furthermore, the complex interactions among hydrogel degradation, cellular behavior, and host tissue responses remain incompletely understood. Recent studies on MAP hydrogel systems have shown that scaffold degradability and cell–material interactions can significantly influence cellular functions and tissue regeneration outcomes.^113^ Similarly, microporous particle-based hydrogels have demonstrated promising performance in tissue engineering and regenerative medicine applications, yet the dynamic interplay between material degradation, tissue remodeling, and long-term therapeutic efficacy remains difficult to predict.^114^ As a result, achieving reliable control over *in vivo* behavior and therapeutic performance continues to be a major challenge for the development of next-generation hydrogel microsphere platforms.

### 4.2 Safety and biocompatibility considerations

Although hydrogel microspheres are generally fabricated from biocompatible natural or synthetic polymers, their safety profile remains a critical consideration for clinical translation. In addition to the intrinsic properties of the matrix materials, residual crosslinking agents, unreacted monomers, and degradation by-products may influence local tissue responses and long-term biocompatibility. For injectable microsphere systems, particle size distribution, degradation kinetics, and accumulation behavior can further affect biosafety and therapeutic outcomes.

Particular attention is required for cell-laden and bioactive hydrogel microspheres, where fabrication conditions, storage procedures, and implantation environments may influence cell viability, phenotype stability, and immune compatibility. Moreover, although many hydrogel microspheres have demonstrated favorable short-term biocompatibility in preclinical studies, comprehensive evaluations of chronic toxicity, biodistribution, foreign-body response, and long-term degradation behavior remain limited. Sterilization also remains challenging for protein- or growth factor-loaded microspheres because commonly used terminal sterilization methods, such as gamma irradiation or heat treatment, may compromise cargo bioactivity and hydrogel structure. In addition, long-term storage of cell-laden microspheres frequently requires cold-chain transportation and cryopreservation strategies, which may further increase manufacturing complexity and translational cost. Therefore, systematic safety assessment, standardized biocompatibility testing, and long-term *in vivo* investigations will be essential for accelerating the clinical translation of hydrogel microsphere-based therapeutic platforms.^106,113^

### 4.3 Future perspectives and clinical translation

Future development of hydrogel microspheres should focus not only on enhancing biological functionality but also on facilitating successful clinical translation. Encouragingly, microsphere-based biomaterials have already demonstrated translational feasibility in several biomedical applications. For example, injectable microporous annealed particle (MAP) hydrogels assembled from hydrogel microspheres have shown promising therapeutic outcomes in regenerative medicine, including accelerated wound healing and improved tissue integration.^115^ In aesthetic medicine, commercially successful hyaluronic-acid-based filler platforms such as Restylane and Juvéderm further demonstrate the long-term clinical use, established biosafety profiles, and broad clinical acceptance that injectable hydrogel formulations can achieve.^106^ In interventional oncology, HepaSphere and Embozene microspheres have obtained regulatory approval for transarterial embolization, providing concrete examples of successful commercialization and clinical implementation of microsphere-based biomaterials.^116^

In the current clinical translation landscape, hydrogel technology is evolving into intelligent, multifunctional therapeutic platforms. The focus of clinical transformation for treating non-healing wounds like diabetic foot ulcers has centered on “smart responsive hydrogels”. These materials can sense pathological signals in the wound microenvironment—such as high glucose, low pH, or high reactive oxygen species—to achieve on-demand drug release and microenvironment modulation,^117^ thereby addressing the limitations of traditional dressings. Simultaneously, to improve patient compliance and promote deep tissue regeneration, innovative delivery systems like bioinspired adaptable indwelling microneedles are entering the clinical field. These systems overcome the limitations of rigid carriers by enabling the sustained release of bioactive agents (*e.g.*, exosomes).^118^ Furthermore, the application scope of hydrogels has expanded into oncological immunotherapy;^119^ for instance, intracavitary sprayable hydrogels delivering nanoregulators aim to remodel the tumor microenvironment and reverse immunosuppression post-surgery, representing a cutting-edge translational direction in cancer adjuvant therapy.

Despite these advances, the translation of next-generation hydrogel microspheres remains limited. Many emerging systems incorporate bioactive molecules, therapeutic cells, or stimuli-responsive functions, which substantially increase manufacturing complexity and regulatory requirements. Future efforts should therefore focus on establishing standardized characterization methods, reproducible manufacturing protocols, and robust quality-control criteria to ensure product consistency and safety.

Regulatory considerations will also play an increasingly important role in clinical implementation. Depending on jurisdiction, intended application, and primary mode of action, hydrogel microspheres may be classified as medical devices, drug-delivery systems, cell carriers, or combination products, each requiring distinct regulatory evaluation pathways.^120^ Sterilization strategy represents another critical translational consideration, as commonly used approaches such as gamma irradiation and ethylene oxide sterilization may alter polymer chain length, crosslink density, or mechanical properties, whereas aseptic processing can better preserve material integrity but imposes more stringent manufacturing requirements.^121^ In addition, cell-laden hydrogel microspheres introduce unique logistical challenges associated with storage and distribution. Cell-based therapeutic products often require refrigerated short-term storage or cryogenic preservation below −130 °C, frequently involving liquid-nitrogen-based storage at −196 °C to maintain long-term viability and functionality.^122^ Similar challenges have been highlighted across multiple emerging therapeutic platforms, where manufacturing scalability, product characterization, quality control, and regulatory harmonization are regarded as essential prerequisites for successful clinical translation.^116,120^ Continued collaboration among researchers, clinicians, industry stakeholders, and regulatory agencies will therefore be critical for advancing hydrogel microspheres from laboratory research to routine clinical use.

Looking ahead, the field is expected to move toward more programmable and multifunctional hydrogel microspheres. Stimuli-responsive chemistries, hybrid nano–micro architectures, image-trackable materials, and modular microsphere assembly strategies are likely to expand therapeutic precision. At the same time, advances in microfluidics, lithography, and scalable droplet engineering may improve reproducibility and enable better control over shape, compartmentalization, and cargo distribution. By combining these advances with rigorous safety evaluation and translationally relevant disease models, hydrogel microspheres may evolve from simple carriers into dynamic therapeutic microenvironments for precision medicine and regenerative nanobiotechnology. Moreover, different biomedical applications may face distinct regulatory pathways. Injectable dermal fillers, drug delivery systems, and cell-based therapeutic microspheres may require substantially different safety evaluation standards and clinical approval processes, which should be considered during early-stage material design.

## 5. Conclusions

Hydrogels and hydrogel microspheres have become versatile biomaterial platforms for precision biomedicine, combining hydrated polymer networks with tunable chemistry, mechanics, degradability and biointerface properties. Compared with bulk hydrogels, microsphere-based systems offer improved injectability, interfacial activity, modular assembly and microporous scaffold formation, making them particularly attractive for localized drug delivery, cell transplantation and tissue regeneration.

The biomedical performance of hydrogel microspheres is closely governed by both network design and fabrication strategy. Physical or chemical crosslinking determines matrix stability, responsiveness and degradation, whereas emulsion polymerization, electrospraying, microfluidics and photolithography control particle size, uniformity, morphology and cargo preservation. Therefore, rational platform design requires matching polymer chemistry and manufacturing route to the intended biological function.

The clinical translation of hydrogel microspheres hinges on surmounting critical bottlenecks in scalable manufacturing, sterilization, long-term storage, biosafety, degradation controllability, and reproducible *in vivo* performance. Leveraging advances in programmable network chemistry, precision microfabrication, and translational bioengineering, these microcarriers are poised to transition from passive delivery vehicles into dynamic therapeutic microenvironments—enabling precision drug delivery, regenerative medicine, and next-generation biomaterial therapies.

Unlike previous reviews that focus narrowly on hydrogel chemistry, fabrication techniques, or specific applications in isolation, this work offers an integrated framework that bridges network engineering, fabrication precision, structure–property relationships, and translational functionality. By systematically correlating material chemistry with microscale processing and biological outcomes, we aim to establish rational design principles for advanced microsphere systems.

Importantly, we identify persistent knowledge gaps—including scalable production, long-term biosafety, programmable degradation, and multifunctional therapeutic integration—whose resolution is imperative for advancing hydrogel microspheres from bench to clinically relevant precision medicine platforms.

## Author contributions

Y. C. S.: visualization, writing—original draft, writing—review and editing. Z. X. X.: visualization, writing—original draft. X. Y. Z.: writing—original draft, writing—review and editing. Z. Q. S.: funding acquisition, supervision, writing—review and editing. Q. B. W.: funding acquisition, supervision, writing—review and editing.

## Conflicts of interest

There are no conflicts to declare.

## Data Availability

No data was used for the research described in the article.

## References

[cit1] Arora S., Rozon J., Laaser J. E. (2021). Dynamics of Ion Locking in Doubly-Polymerized Ionic Liquids. Macromolecules.

[cit2] Yu H., Zhang J., Yang L., Tian Y., Milne C., Jin P. (2025). *et al.*, MSC-derived exosomes injectable hyaluronic acid hydrogel for enhanced chronic wound healing. J. Controlled Release.

[cit3] Shi G., Su T., Li J., Wang A., Gao G., Tao B. (2025). *et al.*, Biomimetic piezoelectric hydrogel system for energy metabolism reprogramming in spinal cord injury repair. Theranostics.

[cit4] Scarton D. V., Coogan A. B., Touma P. M., Tulun E. O., Harrison K. A., Buchen J. (2026). *et al.*, Design and Characterization of DX-Tile DNA Nanostar-Based Hydrogels. Adv. Sci..

[cit5] Zhang F., Zhang H., Wang S., Gao M., Du K., Chen X. (2025). *et al.*, A dynamically phase-adaptive regulating hydrogel promotes ultrafast anti-fibrotic wound healing. Nat. Commun..

[cit6] de Lau W. B. M., Wijnakker J. J. A. P. M., van Son G. J. F., Krueger D., Wang D., Abendroth M. S. (2025). *et al.*, A single-chain derivative of an integrin-activating antibody potentiates organoid growth in Matrigel and collagen hydrogels. Nat. Biotechnol..

[cit7] Arora S., Mao C. (2023). Light-regulated RNA interference induced by p-hydroxyphenacyl-modified siRNA in mammalian cells. Nucleosides, Nucleotides Nucleic Acids.

[cit8] Zhu Y., Xiu Z., Jiang X., Zhang H., Li X., Feng Y. (2025). *et al.*, Injectable hydrogels with ROS-triggered drug release enable the co-delivery of antibacterial agent and anti-inflammatory nanoparticle for periodontitis treatment. J. Nanobiotechnol..

[cit9] Lin Y., Wu H., Wang J., He W., Hou J., Martin V. T. (2025). *et al.*, Nicotinamide Adenine Dinucleotide-Loaded Lubricated Hydrogel Microspheres with a Three-Pronged Approach Alleviate Age-Related Osteoarthritis. ACS Nano.

[cit10] Yang B., Li Z., Yang Z., Zhao P., Lin S., Wu J. (2025). *et al.*, Recapitulating hypoxic metabolism in cartilaginous organoids via adaptive cell-matrix interactions enhances histone lactylation and cartilage regeneration. Nat. Commun..

[cit11] Liu J., Kang J., Zou T., Hu M., Zhang Y., Lin S. (2025). *et al.*, Functional cobalt-doped hydrogel scaffold enhances concurrent vascularization and neurogenesis. J. Nanobiotechnol..

[cit12] Verma N., Arora S., Singh A. K., Kumar A. (2025). Extracellular Vesicle-Associated miRNAs in Cornea Health and Disease: Diagnostic Potential and Therapeutic Implications. Targets.

[cit13] Sun Z., Zheng Q., Zhang Y., Bai C., Wang F., Yang P. (2025). *et al.*, Hydrogel Loaded with Aminoethyl Anisamide-Modified Exosomes Attenuates Hepatic Fibrosis by Targeting Activated Hepatic Stellate Cells. ACS Nano.

[cit14] Verma N., Arora S., Singh A. K., Ahmed J. (2025). Unlocking the potential of exosomes ‘extracellular vesicles’: drug delivery advancements and therapeutics in ocular diseases. RSC Pharm..

[cit15] Ho T.-C., Chang C.-C., Chan H.-P., Chung T.-W., Shu C.-W., Chuang K.-P. (2022). *et al.*, Hydrogels: Properties and Applications in Biomedicine. Molecules.

[cit16] He Z., Hu P., Li Z., Mao K., Zheng J., Yang C.-Y. (2025). *et al.*, Self-assembled hybrid hydrogel microspheres create a bone marrow-mimicking niche for bone regeneration. Bioact. Mater..

[cit17] Chen J., Yan X., Nie L., Zhou S., Ji P., Zhang H. (2025). Injectable hydrogel microsphere orchestrates immune regulation and bone regeneration via sustained release of calcitriol. Mater. Today Bio.

[cit18] Huo Z., Arora S., Kong V. A., Myrga B. J., Statt A., Laaser J. E. (2023). Effect of Polymer Composition and Morphology on Mechanochemical Activation in Nanostructured Triblock Copolymers. Macromolecules.

[cit19] Eagland D., Crowther N. J., Butler C. J. (1994). Complexation between polyoxyethylene and polymethacrylic acid—the importance of the molar mass of polyoxyethylene. Eur. Polym. J..

[cit20] Dumont C. M., Carlson M. A., Munsell M. K., Ciciriello A. J., Strnadova K., Park J. (2019). *et al.*, Aligned hydrogel tubes guide regeneration following spinal cord injury. Acta Biomater..

[cit21] Hu Z., Ma C., Rong X., Zou S., Liu X. (2018). Immunomodulatory ECM-like Microspheres for Accelerated Bone Regeneration in Diabetes Mellitus. ACS Appl. Mater. Interfaces.

[cit22] Kucharska M., Walenko K., Butruk B., Brynk T., Heljak M., Ciach T. (2010). Fabrication and characterization of chitosan microspheres agglomerated scaffolds for bone tissue engineering. Mater. Lett..

[cit23] Xu M., Liao J., Li J., Shi Y., Zhang Z., Fu Y. (2025). *et al.*, Elastic Nanoparticle-Reinforced, Conductive Structural Color Hydrogel With Super Stretchability, Self-Adhesion, Self-Healing as Electrical/Optical Dual-Responsive Visual Electronic Skins. Exploration.

[cit24] Arora S., Verma N. (2026). Streamlining the Removal of Per- and Polyfluoroalkyl Substances (PFAS) From Environment: A Comprehensive Approach. ChemistrySelect.

[cit25] Pupkaite J., Rosenquist J., Hilborn J., Samanta A. (2019). Injectable Shape-Holding Collagen Hydrogel for Cell Encapsulation and Delivery Cross-linked Using Thiol-Michael Addition Click Reaction. Biomacromolecules.

[cit26] Li K. W., Cen L., Zhou C., Zhang A. K., Yao F., Tan L. H. (2016). *et al.*, Well-Defined Poly(ethylene glycol) Hydrogels with Enhanced Mechanical Performance Prepared by Thermally Induced Copper-Catalyzed Azide–Alkyne Cycloaddition. Macromol. Mater. Eng..

[cit27] Xin S., Chimene D., Garza J. E., Gaharwar A. K., Alge D. L. (2019). Clickable PEG hydrogel microspheres as building blocks for 3D bioprinting. Biomater. Sci..

[cit28] Gyarmati B., Némethy Á., Szilágyi A. (2013). Reversible disulphide formation in polymer networks: A versatile functional group from synthesis to applications. Eur. Polym. J..

[cit29] Xu J., Liu Y., Hsu S.-H. (2019). Hydrogels Based on Schiff Base Linkages for Biomedical Applications. Molecules.

[cit30] Wang Y. J., Zhang X. N., Song Y., Zhao Y., Chen L., Su F. (2019). *et al.*, Ultrastiff and Tough Supramolecular Hydrogels with a Dense and Robust Hydrogen Bond Network. Chem. Mater..

[cit31] Zhang X., Guo X., Yang S., Tan S., Li X., Dai H. (2009). *et al.*, Double-network hydrogel with high mechanical strength prepared from two biocompatible polymers. J. Appl. Polym. Sci..

[cit32] Agrawal S. K., Sanabria-DeLong N., Tew G. N., Bhatia S. R. (2008). Structural Characterization of PLA−PEO−PLA Solutions and Hydrogels: Crystalline vs. Amorphous PLA Domains. Macromolecules.

[cit33] Wegner S. V., Schenk F. C., Witzel S., Bialas F., Spatz J. P. (2016). Cobalt Cross-Linked Redox-Responsive PEG Hydrogels: From Viscoelastic Liquids to Elastic Solids. Macromolecules.

[cit34] Lopez-Perez P. M., da Silva R. M. P., Strehin I., Kouwer P. H. J., Leeuwenburgh S. C. G., Messersmith P. B. (2017). Self-Healing Hydrogels Formed by Complexation between Calcium Ions and Bisphosphonate-Functionalized Star-Shaped Polymers. Macromolecules.

[cit35] Shibayama M. (2017). Exploration of Ideal Polymer Networks. Macromol. Symp..

[cit36] Liu S., Li K., Hussain I., Oderinde O., Yao F., Zhang J. (2018). *et al.*, A Conductive Self-Healing Double Network Hydrogel with Toughness and Force Sensitivity. Chem.–Eur. J..

[cit37] Zhang Y.-L., Chang R., Duan H.-Z., Chen Y.-X. (2020). Metal ion and light sequentially induced sol–gel–sol transition of a responsive peptide-hydrogel. Soft Mater..

[cit38] Liu S., Dong M., Zhang Z., Fu G. (2017). High elasticity, strength, and biocompatible amphiphilic hydrogel via click chemistry and ferric ion coordination. Polym. Adv. Technol..

[cit39] Liu S., Oderinde O., Hussain I., Yao F., Fu G. (2018). Dual ionic cross-linked double network hydrogel with self-healing, conductive, and force sensitive properties. Polymer.

[cit40] Coviello T., Grassi M., Rambone G., Santucci E., Carafa M., Murtas E. (1999). *et al.*, Novel hydrogel system from scleroglucan: synthesis and characterization. J. Controlled Release.

[cit41] Kolb H. C., Finn M. G., Sharpless K. B. (2001). Click Chemistry: Diverse Chemical Function from a Few Good Reactions. Angew. Chem., Int. Ed..

[cit42] Arora S., Liang J., Fullerton-Shirey S. K., Laaser J. E. (2020). Triggerable Ion Release in Polymerized Ionic Liquids Containing Thermally Labile Diels–Alder Linkages. ACS Mater. Lett..

[cit43] Arora S., Verma N. (2024). A review: advancing organic electronics through the lens of ionic liquids and polymerized ionic liquids. RSC Appl. Polym..

[cit44] Ren Y., Guo J., Liu Z., Sun Z., Wu Y., Liu L. (2019). *et al.*, Ionic liquid–based click-ionogels. Sci. Adv..

[cit45] Truong V. X., Tsang K. M., Forsythe J. S. (2017). Nonswelling Click-Cross-Linked Gelatin and PEG Hydrogels with Tunable Properties Using Pluronic Linkers. Biomacromolecules.

[cit46] Qayyum A. S., Jain E., Kolar G., Kim Y., Sell S. A., Zustiak S. P. (2017). Design of electrohydrodynamic sprayed polyethylene glycol hydrogel microspheres for cell encapsulation. Biofabrication.

[cit47] Utama R. H., Tan V. T. G., Tjandra K. C., Sexton A., Nguyen D. H. T., O’Mahony A. P. (2021). *et al.*, A Covalently Crosslinked Ink for Multimaterials Drop-on-Demand 3D Bioprinting of 3D Cell Cultures. Macromol. Biosci..

[cit48] Hunckler M. D., Medina J. D., Coronel M. M., Weaver J. D., Stabler C. L., García A. J. (2019). Linkage Groups within Thiol–Ene Photoclickable PEG Hydrogels Control In Vivo Stability. Adv. Healthcare Mater..

[cit49] Yu J., Chen F., Wang X., Dong N., Lu C., Yang G. (2016). *et al.*, Synthesis and characterization of MMP degradable and maleimide cross-linked PEG hydrogels for tissue engineering scaffolds. Polym. Degrad. Stab..

[cit50] Sharma P. K., Singh Y. (2019). Glyoxylic Hydrazone Linkage-Based PEG Hydrogels for Covalent Entrapment and Controlled Delivery of Doxorubicin. Biomacromolecules.

[cit51] Gould S. T., Anseth K. S. (2016). Role of cell-matrix interactions on VIC phenotype and tissue deposition in 3D PEG hydrogels. J. Tissue Eng. Regen. Med..

[cit52] Schweller R. M., Wu Z. J., Klitzman B., West J. L. (2017). Stiffness of Protease Sensitive and Cell Adhesive PEG Hydrogels Promotes Neovascularization In Vivo. Ann. Biomed. Eng..

[cit53] Zhang M., Song C.-C., Du F.-S., Li Z.-C. (2017). Supersensitive Oxidation-Responsive Biodegradable PEG Hydrogels for Glucose-Triggered Insulin Delivery. ACS Appl. Mater. Interfaces.

[cit54] Vats K., Marsh G., Harding K., Zampetakis I., Waugh R. E., Benoit D. S. W. (2017). Nanoscale physicochemical properties of chain- and step-growth polymerized PEG hydrogels affect cell-material interactions. J. Biomed. Mater. Res., Part A.

[cit55] Stenekes R. J. H., Franssen O., van Bommel E. M. G., Crommelin D. J. A., Hennink W. E. (1998). The Preparation of Dextran Microspheres in an All-Aqueous System: Effect of the Formulation Parameters on Particle Characteristics. Pharm. Res..

[cit56] Xu Q., Hashimoto M., Dang T. T., Hoare T., Kohane D. S., Whitesides G. M. (2009). *et al.*, Preparation of Monodisperse Biodegradable Polymer Microparticles Using a Microfluidic Flow-Focusing Device for Controlled Drug Delivery. Small.

[cit57] Dendukuri D., Pregibon D. C., Collins J., Hatton T. A., Doyle P. S. (2006). Continuous-flow lithography for high-throughput microparticle synthesis. Nat. Mater..

[cit58] Pancholi K., Ahras N., Stride E., Edirisinghe M. (2008). Novel electrohydrodynamic preparation of porous chitosan particles for drug delivery. J. Mater. Sci.: Mater. Med..

[cit59] Daly A. C., Riley L., Segura T., Burdick J. A. (2019). Hydrogel microparticles for biomedical applications. Nat. Rev. Mater..

[cit60] Leiza J. R. and Meuldijk J., Emulsion Copolymerisation, Process Strategies, Chemistry and Technology of Emulsion Polymerisation, 2013, pp. 75–104

[cit61] Liu L., Wu Q., Ma X., Xiong D., Gong C., Qian Z. (2013). *et al.*, Camptothecine encapsulated composite drug delivery system for colorectal peritoneal carcinomatosis therapy: Biodegradable microsphere in thermosensitive hydrogel. Colloids Surf., B.

[cit62] Zhou M., Wang P., Song Y., Li H., Luo J., Pan J. (2022). Hybrid hydrogel microspheres loading single-hole hollow imprinted particles for fast and selective uptake of 2′-deoxyadenosine. Sep. Purif. Technol..

[cit63] Zhou Z.-F., Sun T.-W., Chen F., Zuo D.-Q., Wang H.-S., Hua Y.-Q. (2017). *et al.*, Calcium phosphate-phosphorylated adenosine hybrid microspheres for anti-osteosarcoma drug delivery and osteogenic differentiation. Biomaterials.

[cit64] Greiner A., Wendorff J. H. (2007). Electrospinning: A Fascinating Method for the Preparation of Ultrathin Fibers. Angew. Chem., Int. Ed..

[cit65] Sideris E., Griffin D. R., Ding Y., Li S., Weaver W. M., Di Carlo D. (2016). *et al.*, Particle Hydrogels Based on Hyaluronic
Acid Building Blocks. ACS Biomater. Sci. Eng..

[cit66] Wang B., Prinsen P., Wang H., Bai Z., Wang H., Luque R. (2017). *et al.*, Macroporous materials: microfluidic fabrication, functionalization and applications. Chem. Soc. Rev..

[cit67] Kim P.-H., Yim H.-G., Choi Y.-J., Kang B.-J., Kim J., Kwon S.-M. (2014). *et al.*, Injectable multifunctional microgel encapsulating outgrowth endothelial cells and growth factors for enhanced neovascularization. J. Controlled Release.

[cit68] Gu Z., Dang T. T., Ma M., Tang B. C., Cheng H., Jiang S. (2013). *et al.*, Glucose-Responsive Microgels Integrated with Enzyme Nanocapsules for Closed-Loop Insulin Delivery. ACS Nano.

[cit69] Pittermannová A., Ruberová Z., Zadražil A., Bremond N., Bibette J., Štěpánek F. (2016). Microfluidic fabrication of composite hydrogel microparticles in the size range of blood cells. RSC Adv..

[cit70] Thorsen T., Roberts R. W., Arnold F. H., Quake S. R. (2001). Dynamic Pattern Formation in a Vesicle-Generating Microfluidic Device. Phys. Rev. Lett..

[cit71] Seo M., Nie Z., Xu S., Mok M., Lewis P. C., Graham R. (2005). *et al.*, Continuous Microfluidic Reactors for Polymer Particles. Langmuir.

[cit72] Nie Z., Xu S., Seo M., Lewis P. C., Kumacheva E. (2005). Polymer Particles with Various Shapes and Morphologies Produced in Continuous Microfluidic Reactors. J. Am. Chem. Soc..

[cit73] De Geest B. G., Urbanski J. P., Thorsen T., Demeester J., De Smedt S. C. (2005). Synthesis of Monodisperse Biodegradable Microgels in Microfluidic Devices. Langmuir.

[cit74] Liang J., Xu K., Arora S., Laaser J. E., Fullerton-Shirey S. K. (2020). Ion-Locking in Solid Polymer Electrolytes for Reconfigurable Gateless Lateral Graphene p–n Junctions. Materials.

[cit75] Nichol J. W., Koshy S. T., Bae H., Hwang C. M., Yamanlar S., Khademhosseini A. (2010). Cell-laden microengineered gelatin methacrylate hydrogels. Biomaterials.

[cit76] Helgeson M. E., Chapin S. C., Doyle P. S. (2011). Hydrogel microparticles from lithographic processes: Novel materials for fundamental and applied colloid science. Curr. Opin. Colloid Interface Sci..

[cit77] Chung S. E., Park W., Shin S., Lee S. A., Kwon S. (2008). Guided and fluidic self-assembly of microstructures using railed microfluidic channels. Nat. Mater..

[cit78] Lee S. A., Chung S. E., Park W., Lee S. H., Kwon S. (2009). Three-dimensional fabrication of heterogeneous microstructures using soft membrane deformation and optofluidic maskless lithography. Lab Chip.

[cit79] Laza S. C., Polo M., Neves A. A. R., Cingolani R., Camposeo A., Pisignano D. (2012). Two-Photon Continuous Flow Lithography. Adv. Mater..

[cit80] Nielson R., Kaehr B., Shear J. B. (2009). Microreplication and Design of Biological Architectures Using Dynamic-Mask Multiphoton Lithography. Small.

[cit81] Dendukuri D., Gu S. S., Pregibon D. C., Hatton T. A., Doyle P. S. (2007). Stop-flow lithography in a microfluidic device. Lab Chip.

[cit82] Panda P., Ali S., Lo E., Chung B. G., Hatton T. A., Khademhosseini A. (2008). *et al.*, Stop-flow lithography to generate cell-laden microgel particles. Lab Chip.

[cit83] Xiao Z., Chen K., Lin T., Zhao P., Wang H., Su Z. (2024). Versatile hydrogel drug carrier loading with peptide-carbon dots conjugates for efficient targeting and synergistic suppression of breast cancer cell. Mater. Today Commun..

[cit84] Lai W.-F., Susha A. S., Rogach A. L., Wang G., Huang M., Hu W. (2017). *et al.*, Electrospray-mediated preparation of compositionally homogeneous core–shell hydrogel microspheres for sustained drug release. RSC Adv..

[cit85] Han Y., Yang J., Zhao W., Wang H., Sun Y., Chen Y. (2021). *et al.*, Biomimetic injectable hydrogel microspheres with enhanced lubrication and controllable drug release for the treatment of osteoarthritis. Bioact. Mater..

[cit86] Dong X., Chi J., Shao C., Lei L., Yang L., Zhao C. (2021). *et al.*, Multifunctional hydrogel microsphere with reflection in near-infrared region for in vivo pH monitoring and drug release in tumor microenvironment. Chem. Eng. J..

[cit87] Chaturvedi K., Ganguly K., Nadagouda M. N., Aminabhavi T. M. (2013). Polymeric hydrogels for oral insulin delivery. J. Controlled Release.

[cit88] Sajeesh S., Bouchemal K., Marsaud V., Vauthier C., Sharma C. P. (2010). Cyclodextrin complexed insulin encapsulated hydrogel microparticles: An oral delivery system for insulin. J. Controlled Release.

[cit89] Mundargi R. C., Rangaswamy V., Aminabhavi T. M. (2011). Poly(N-vinylcaprolactam-co-methacrylic acid) hydrogel microparticles for oral insulin delivery. J. Microencapsulation.

[cit90] Bell C. L., Peppas N. A. (1996). Water, solute and protein diffusion in physiologically responsive hydrogels of poly(methacrylic acid-g-ethylene glycol). Biomaterials.

[cit91] Censi R., Di Martino P., Vermonden T., Hennink W. E. (2012). Hydrogels for protein delivery in tissue engineering. J. Controlled Release.

[cit92] Li J., Mooney D. J. (2016). Designing hydrogels for controlled drug delivery. Nat. Rev. Mater..

[cit93] Mealy J. E., Chung J. J., Jeong H. H., Issadore D., Lee D., Atluri P. (2018). *et al.*, Injectable Granular Hydrogels with Multifunctional Properties for Biomedical Applications. Adv. Mater..

[cit94] Madl C. M., Heilshorn S. C., Blau H. M. (2018). Bioengineering strategies to accelerate stem cell therapeutics. Nature.

[cit95] Prince E., Kumacheva E. (2019). Design and applications of man-made biomimetic fibrillar hydrogels. Nat. Rev. Mater..

[cit96] Seliktar D. (2012). Designing Cell-Compatible Hydrogels for Biomedical Applications. Science.

[cit97] Hamilton M., Harrington S., Dhar P., Stehno-Bittel L. (2021). Hyaluronic Acid Hydrogel Microspheres for Slow Release Stem Cell Delivery. ACS Biomater. Sci. Eng..

[cit98] Zhang R., Xie L., Wu H., Yang T., Zhang Q., Tian Y. (2020). *et al.*, Alginate/laponite hydrogel microspheres co-encapsulating dental pulp stem cells and VEGF for endodontic regeneration. Acta Biomater..

[cit99] Zhao X., Liu S., Yildirimer L., Zhao H., Ding R., Wang H. (2016). *et al.*, Injectable Stem Cell-Laden Photocrosslinkable Microspheres Fabricated Using Microfluidics for Rapid Generation of Osteogenic Tissue Constructs. Adv. Funct. Mater..

[cit100] Annamalai R. T., Hong X., Schott N. G., Tiruchinapally G., Levi B., Stegemann J. P. (2019). Injectable osteogenic microtissues containing mesenchymal stromal cells conformally fill and repair critical-size defects. Biomaterials.

[cit101] Cha C., Oh J., Kim K., Qiu Y., Joh M., Shin S. R. (2014). *et al.*, Microfluidics-Assisted Fabrication of Gelatin-Silica Core–Shell Microgels for Injectable Tissue Constructs. Biomacromolecules.

[cit102] Feyen D. A. M., Gaetani R., Deddens J., van Keulen D., van Opbergen C., Poldervaart M. (2016). *et al.*, Gelatin Microspheres as Vehicle for Cardiac Progenitor Cells Delivery to the Myocardium. Adv. Healthcare Mater..

[cit103] Collins M. N., Birkinshaw C. (2013). Hyaluronic acid based scaffolds for tissue engineering—A review. Carbohydr. Polym..

[cit104] Xin S., Wyman O. M., Alge D. L. (2018). Assembly of PEG Microgels into Porous Cell-Instructive 3D Scaffolds via Thiol-Ene Click Chemistry. Adv. Healthcare Mater..

[cit105] Truong N. F., Kurt E., Tahmizyan N., Lesher-Pérez S. C., Chen M., Darling N. J. (2019). *et al.*, Microporous annealed particle hydrogel stiffness, void space size, and adhesion properties impact cell proliferation, cell spreading, and gene transfer. Acta Biomater..

[cit106] Gilbert E., Hui A., Waldorf H. A. (2012). The Basic Science of Dermal Fillers: Past and Present Part I: Background and Mechanisms of Action. J. Drugs Dermatol..

[cit107] Tang Q., Lu B., He J., Chen X., Fu Q., Han H. (2022). *et al.*, Exosomes-loaded thermosensitive hydrogels for corneal epithelium and stroma regeneration. Biomaterials.

[cit108] Xu Y., Wei C., Ma L., Zhao L., Li D., Lin Y. (2025). *et al.*, 3D mesenchymal stem cell exosome-functionalized hydrogels for corneal wound healing. J. Controlled Release.

[cit109] Yang M., Chen X., Chen Z., Zhao N., Zeng Z., Huang X. (2025). *et al.*, Thermoresponsive antioxidant metal-free carbon nanodot hydrogel: An effective therapeutic approach for ocular surface disease. Sci. Adv..

[cit110] Wang W., Hua Q., Xia X., Li J., Liu C., Wang B. (2026). Microfluidic Gd3+-chelated hydrogel microspheres enable MRI-visible, image-guided photothermal chemotherapy for pancreatic cancer. Colloids Surf., B.

[cit111] Lu P., Yuan H., Wang G., Dong Y., Zhao R., Man J. (2025). *et al.*, Programmatically activated DNA hydrogel microcapsules for precision therapy in inflammatory bowel disease. Theranostics.

[cit112] Rose J. C., Cámara-Torres M., Rahimi K., Köhler J., Möller M., De Laporte L. (2017). Nerve Cells Decide to Orient inside an Injectable Hydrogel with Minimal Structural Guidance. Nano Lett..

[cit113] Xin S., Gregory C. A., Alge D. L. (2020). Interplay between degradability and integrin signaling on mesenchymal stem cell function within poly(ethylene glycol) based microporous annealed particle hydrogels. Acta Biomater..

[cit114] Isaac A., Jivan F., Xin S., Hardin J., Luan X., Pandya M. (2019). *et al.*, Microporous Bio-orthogonally Annealed Particle Hydrogels for Tissue Engineering and Regenerative Medicine. ACS Biomater. Sci. Eng..

[cit115] Griffin D. R., Weaver W. M., Scumpia P. O., Di Carlo D., Segura T. (2015). Accelerated wound healing by injectable microporous gel scaffolds assembled from annealed building blocks. Nat. Mater..

[cit116] Pérez-López A., Martín-Sabroso C., Gómez-Lázaro L., Torres-Suárez A. I., Aparicio-Blanco J. (2022). Embolization therapy with microspheres for the treatment of liver cancer: State-of-the-art of clinical translation. Acta Biomater..

[cit117] Chen Y., Wang X., Tao S., Wang Q., Ma P.-Q., Li Z.-B. (2023). *et al.*, Research advances in smart responsive-hydrogel dressings with potential clinical diabetic wound healing properties. Mil. Med. Res..

[cit118] Zhang X., Gan J., Fan L., Luo Z., Zhao Y. (2023). Bioinspired Adaptable Indwelling Microneedles for Treatment of Diabetic Ulcers. Adv. Mater..

[cit119] Dong Y., Zhang J., Wang Y., Zhang Y., Rappaport D., Yang Z. (2023). *et al.*, Intracavitary Spraying of Nanoregulator-Encased Hydrogel Modulates Cholesterol Metabolism of Glioma-Supportive Macrophage for Postoperative Glioblastoma Immunotherapy. Adv. Mater..

[cit120] Verma N., Arora S. (2025). Navigating the Global Regulatory Landscape for Exosome-Based Therapeutics: Challenges, Strategies, and Future Directions. Pharmaceutics.

[cit121] Bhatnagar D., Dube K., Damodaran V. B., Subramanian G., Aston K., Halperin F. (2016). *et al.*, Effects of Terminal Sterilization on PEG-Based Bioresorbable Polymers Used in Biomedical Applications. Macromol. Mater. Eng..

[cit122] Meneghel J., Kilbride P., Morris G. J. (2020). Cryopreservation as a Key Element in the Successful Delivery of Cell-Based Therapies—A Review. Front. Med..

